# Soil–plant interactions regulating the mobility and acquisition of Zn, Fe, Se, and I: implications for crop biofortification strategies

**DOI:** 10.3389/fpls.2026.1968376

**Published:** 2026-10-26

**Authors:** Shirley Zevallos, Pedro Gutierrez-Vilchez, Anyela Quispe, Cynthia de Oliveira, Everton Geraldo de Morais, Guilherme Lopes, Luiz Roberto Guimarães Guilherme

**Affiliations:** 1 Department of Soil Science, Universidad Nacional Agraria La Molina, Lima, Peru; 2 Department of Soil Science, Federal University of Lavras, Lavras, Minas Gerais, Brazil; 3 Department of Natural Sciences and Earth, Minas Gerais State University (UEMG), Divinópolis, Minas Gerais, Brazil

**Keywords:** agronomic biofortification, iron, micronutrient availability, micronutrient transport, nutrient acquisition, selenium, soil–plantsoil–plant continuum, zinc

## Abstract

Micronutrient deficiencies, particularly those involving zinc (Zn), iron (Fe), selenium (Se), and iodine (I), remain major constraints to human nutrition and food security. Crop biofortification offers a sustainable approach to increasing the concentration and bioavailability of these elements in staple foods. Still, its effectiveness depends on the processes regulating their behavior across the soil–plantsoil–plant continuum. This structured narrative review synthesizes current evidence on the edaphic, rhizospheric, physiological, and molecular mechanisms controlling Zn, Fe, Se, and I mobility, acquisition, transport, redistribution, storage, and accumulation in edible plant organs. Literature searches were conducted in Scopus, Web of Science, PubMed, and Google Scholar, focusing primarily on studies published between 2021 and 18 March 2026, while retaining foundational research required to explain key mechanisms. The evidence indicates that parent material, texture, cation exchange capacity, pH, redox potential, organic matter, and nutrient interactions govern micronutrient speciation and availability. Plants respond through rhizosphere acidification, chelator and organic acid release, microbial associations, and coordinated transporter networks. Zinc and Fe homeostasis involves ZIP/IRT, HMA, YSL, NRAMP, MTP, VIT, and related transport systems. In contrast, Se largely shares sulfate and phosphate pathways, and iodine appears to use anion transport systems associated with chloride. Agronomic fertilization can rapidly increase micronutrient concentrations, while breeding, biotechnology, and gene editing can improve uptake, remobilization, and storage. The most promising strategies integrate soil diagnosis, appropriate nutrient sources and application methods, genotype selection, microbial processes, bioavailability, and food-system considerations. Such integration is essential for producing nutritionally improved crops without compromising yield, environmental sustainability, or consumer acceptance.

## 1 Review methodology

### 1.1 Review design

This study was conducted as a structured narrative review supported by a structured literature search to synthesize current knowledge on the soil–plant mechanisms regulating the mobility, availability, acquisition, transport, redistribution, and accumulation of zinc (Zn), iron (Fe), selenium (Se), and iodine (I), as well as their implications for agronomic, genetic, biotechnological, and integrated crop biofortification strategies.

The central aim of this review was to integrate current evidence across the soil–plant continuum to identify the edaphic, rhizospheric, physiological and molecular processes that determine micronutrient acquisition and accumulation in plant organs. The review was guided by the following research questions: (i) Which soil physicochemical and geochemical factors regulate the mobility of Zn, Se, Fe and I? (ii) Which rhizosphere processes and root acquisition mechanisms control the uptake of these micronutrients? (iii) Which transport, redistribution, homeostasis, and storage mechanisms determine their accumulation in edible plant organs? and (iv) How can knowledge of these mechanisms be integrated into agronomic, genetic, biotechnological, and combined biofortification strategies?

The review followed six sequential stages: (i) definition of the scope and research questions; (ii) structured identification of relevant literature; (iii) relevance assessment and study selection; (iv) data extraction and thematic organization; (v) qualitative evidence synthesis; and (vi) interpretation and reporting. The methodological workflow is summarized in **Figure 1**.

**Figure 1:**
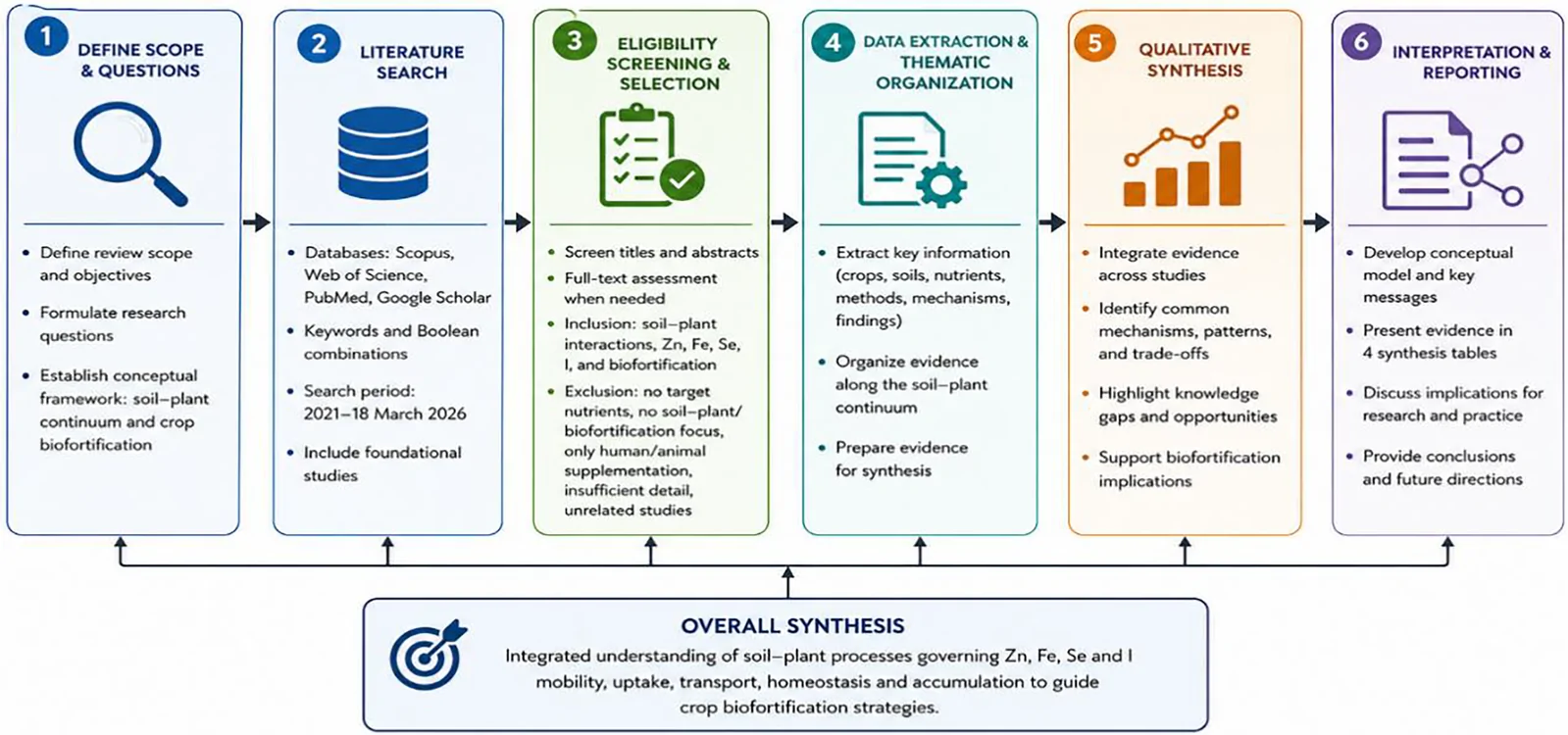
Methodological workflow of the structured narrative review.

### 1.2 Literature search strategy

The literature search was conducted on Scopus, Web of Science, PubMed, and Google Scholar and was completed on 18 March 2026. These databases were selected because they provide broad coverage of peer-reviewed literature in soil science, plant nutrition, agronomy, plant physiology, molecular biology, crop breeding, biotechnology, and human nutrition.

The search strategy combined terms related to soil–plant interactions, the four target micronutrients, nutrient mobility and bioavailability, root uptake, rhizosphere processes, plant transport systems, homeostasis, and crop biofortification. Representative terms included zinc, iron, selenium, iodine, soil–plant interaction, soil availability, rhizosphere, root uptake, plant transporters, xylem transport, phloem mobility, homeostasis, agronomic biofortification, genetic biofortification, biotechnological biofortification, foliar fertilization, soil fertilization, conventional breeding, and gene editing

Search terms were combined using Boolean operators according to the syntax required by each database. The representative search equation was:

(“zinc” OR “iron” OR “selenium” OR “iodine”) AND (“soil–plant interaction” OR “rhizosphere” OR “soil availability” OR “root uptake” OR “transporters” OR “homeostasis”) AND (“biofortification” OR “agronomic biofortification” OR “genetic biofortification” OR “biotechnological biofortification” OR “crop nutrition”).

The primary investigation involved the exploration of publications which were released in the period starting from 2021 to 18 March 2026. However, foundational studies were involved in the process given that they contained initial evidence of micronutrient chemistry, adsorption and desorption, nutrient transporters, vascular transport, intracellular sequestration, plant homeostasis, and the creation of crop biofortification in the past and across centuries.

### 1.3 Eligibility criteria and study selection

Studies were included if they provided experimental, physiological, molecular, agronomic, or mechanistic evidence relevant to the soil–plant continuum of Zn, Fe, Se, or I, or to crop biofortification. The studies that evaluated food crops, especially cereals, potatoes, legumes, vegetables, or other staple crops were prioritized. However, studies involving non-food plants or model species were also considered if they provided valuable information regarding, for example, the function of transporters, competition for nutrients, synthesis of chelators, homeostasis, sequestration, and other tolerance mechanisms.

The sources of evidence were made up of original research articles and review papers. Supplemental literature such as technical reports, institutional publications, books, and book chapters were also considered when they offered scientific information that was deemed insufficiently represented in the most recently published peer-reviewed studies.

Publications were omitted if they: (i) were not related to Zn, Fe, Se, or I; (ii) did not include a soil–plant continuum or crop biofortification aspect; (iii) only pertained to human or animal supplementation; (iv) had insufficient scientific or methodological data; or (v) were irrelevant to review goals.

Titles and abstracts were initially assessed for relevance, followed by full-text evaluation when necessary. Because detailed records of the numbers of studies identified, screened, excluded, and retained were not systematically documented, this study is presented as a structured narrative review rather than a formal systematic review. Study selection was therefore guided by the predefined scope, eligibility criteria, and relevance of each publication to the soil–plant mechanisms and biofortification themes addressed in this review.

### 1.4 Data extraction and thematic organization

Data were obtained through hand extraction from relevant literature. There were many variables that were recorded including years of publication, country of publication, crop species information, experimental site descriptions, main micronutrients, soils used, quality of fertilizer, method of application, dosage, plant materials used, techniques adopted, and conclusions reached by the authors.

The data were classified based on the soil–plantsoil–plant continuum, namely: (i) soil factors responsible for micronutrient mobility and availability; (ii) rhizosphere processes involved in acquisition of micronutrients by plants; (iii) intake, translocation, assimilation, upkeep, storage of plant nutrients; and (iv) various agronomic and biotechnological strategies adopted for biofortification of crops.

The evidence collected is shown through the development of four tables which contain additional information. **Table 1** describes the impacts the main soil parameters (including pH, organic matter content, and so on) have on the presence of zinc, iron, selenium, and iodine in soils. **Table 2** shows a comparative analysis of ways plants accumulate micronutrients. **Table 3** is dedicated to the transport of micronutrients in plants and their storage. **Table 4** presents an evidence matrix regarding agronomic, genetic, and integrated biofortification practices.

**Table 1:** Directional effects of major soil factors on the mobility and bioavailability of Zn, Fe, Se and I.

Factor	General mechanism	Zn	Fe	Se	I	Key references
Parent material and mineralogy	Controls total reserves and weathering inputs	Low in quartz-rich sands; associated with Zn minerals	Fe oxides are major reservoirs and sorbents	Shales may be enriched; granites often poor	Atmospheric inputs dominate; Fe/Al minerals retain IO_3_^-^	Alloway, 2008; Schiavon et al., 2020; Dinh et al., 2018; Shetaya et al., 2012; Dai et al., 2009
Texture and CEC	Controls surface area and ionic retention	Clay/silt retain more; sands often deficient	High oxide/clay surfaces increase retention	Clay minerals adsorb oxyanions	SOM and colloids strongly retain I	Hafeez et al., 2013; Praharaj et al., 2021; Taşpınar et al., 2025; Dinh et al., 2018; Dai et al., 2020
Increasing pH	Changes charge, hydrolysis and precipitation	Strong decrease; ZnCO_3_ and Zn(OH)_2_	Very strong decrease; Fe(OH)_3_	Often favors mobile SeO_4_^2-^ under oxidation	IO_3_^-^ may persist; I^-^ adsorption varies	Alloway, 2008; White and Broadley, 2011; Koç and Karayiğit, 2021; Deng et al., 2025; Rakoczy-Lelek et al., 2021
Reducing conditions	Changes oxidation state and mineral dissolution	ZnS may precipitate	Fe^3+^ reduction increases Fe^2+^ solubility	Se^0^/Se^2-^ become poorly mobile	IO_3_^-^ is reduced to I^-^	Alloway, 2008; Schiavon et al., 2020; Deng et al., 2025; Jiang et al., 2024
SOM/humus	Adsorption, complexation and mineralization	Dual role: soluble complexes or immobilization	Chelation may increase transport; sorption may retain Fe	Reservoir released by mineralization or immobilized	Major soil reservoir; rapid organification	Jalal et al., 2024; Colombo et al., 2014; Li et al., 2016; Huang et al., 2026; Dai et al., 2020; Naeem et al., 2026
High P	Competition and insoluble compound formation	Zn–P antagonism and precipitation	Fe–P interactions reduce availability	SeO_3_^2-^ competes with phosphate	Evidence is less clearly established	Sánchez-Rodríguez et al., 2017; Silva-Herrera et al., 2025; Fu et al., 2025

Responses are conceptual and may vary with mineralogy, organic matter, redox regime, nutrient concentration and biological activity.

**Table 2:** Comparative overview of uptake, transport, assimilation and homeostatic mechanisms for Zn, Fe, Se and I in plants.

Element	Absorbed forms	Root uptake	Long-distance transport	Storage and assimilation	Key interaction	Key references
Zn	Zn^2+^; Zn–phytosiderophore complexes	ZIP/IRT, NRAMP, YSL	HMA2/HMA4; nicotianamine complexes	MTP/CDF, CAX, ZIF; vacuoles	Competition with Fe, P, Cu, Cd	Ajeesh Krishna et al., 2020; Kumar et al., 2016; Hamzah Saleem et al., 2022; Pypka et al., 2025; Robe et al., 2025
Fe	Fe^2+^; Fe^3+^–chelates	IRT1; YS1/YSL after reduction or chelation	FRD3–citrate, Fe–NA, YSL	VIT, ferritin, vacuoles/chloroplasts	Zn competition; Fenton toxicity	Molnár et al., 2023; Zhu et al., 2022; Mentewab et al., 2023; Senguttuvel et al., 2023; Nascimento et al., 2022
Se	SeO_4_^2-^; SeO_3_^2-^; H_2_SeO_3_	SULTR1; PHT1; NIP aquaporins	SULTR2/3; amino acid transporters	SeCys, SeMet, MeSeCys; volatilization	Competes with S and P	Trippe and Pilon-Smits, 2021; Zhang and Chu, 2022; Fu et al., 2025; Deng et al., 2025; Nascimento et al., 2022
I	I^-^; IO_3_^-^; organic I	Probable H^+^/anion symporters; CLC, CCC, ALMT, SLAH	Mainly xylem; limited phloem mobility	Vacuolar I^-^ and organic I; CH_3_I volatilization	Shares pathways with Cl^-^	White et al., 2009; De Angeli et al., 2006; Kiferle et al., 2021; Riyazuddin et al., 2023; Nascimento et al., 2022; Silva-Herrera et al., 2025

**Table 3:** Long-distance transport, storage patterns and physiological constraints associated with Zn, Fe, Se and I homeostasis.

Element	Transport form	Key components	Sink/storage	Physiological constraint	Key references
Zn	Xylem as chelated Zn; phloem to sinks	HMA2/HMA4, YSL, NA	Tubers, grains, young tissues; vacuoles	Deficiency <15–20 mg kg^-1^ DW; optimum 30–100; toxicity often 100–700 mg kg^-1^ DW	Ajeesh Krishna et al., 2020; White and Broadley, 2011; Kaur and Garg, 2021.
Fe	Fe^3+^–citrate and Fe–NA complexes	FRD3, LPR1/2, WBC19, YSL	Chloroplasts, ferritin, vacuoles	Excess Fe^2+^ generates ROS through Fenton chemistry	Nascimento et al., 2022; Gao et al., 2023
Se	Selenate in xylem; SeMet remobilized to grains	SULTR2/3, amino acid transporters	Plastids, proteins, methylated compounds	No universal threshold has been established. Tissue Se concentrations >~5 mg kg^-1^ DW are frequently associated with toxicity in non-accumulator crops, although tolerance varies widely among species. Toxicity symptoms have been reported at ~2.0 mg kg^-1^ DW in rice and ~4.9 mg kg^-1^ DW in wheat.	Wang et al., 2025; Akhter et al., 2026
I	Predominantly xylem-driven; limited phloem movement	Putative anion transporters/channels	Roots, leaves, stems; vacuoles; iodinated metabolites	No universal tissue threshold has been established. Iodine phytotoxicity strongly depends on plant species, chemical form, application method, and growth conditions. Concentrations above approximately 10– 100 µM I in nutrient solution may inhibit plant growth depending on the species. Biomass reductions have also been reported at >50 mg I kg^-1^ soil in tomato, 80 mg I kg^-1^ soil in potato, and 5 mg I L^-1^ in hydroponically grown lettuce.	Medrano-Macías et al., 2016; Buturi et al., 2021; Vetési et al., 2022

**Table 4:** Detailed evidence matrix of agronomic and genetic crop biofortification approaches.

Biofortification approach	Citation	Crop	Country	Experimental environment	Nutrient source and dose	Application timing and method	Variables evaluated	Main quantitative results	Scientific interpretation and limitations
Agronomic biofortification	Cardoso et al. (2026)	Upland Rice	Brazil	Two field experiments Soil Zn availability: 2.70 and 1.20 mg dm^-3^	Zinc sulfate (ZnSO_4_·7H_2_O) at 3 kg ha^-1^	Foliar spray at reproductive stages R3 and R7	Yield, Zn content in paddy/brown/white rice, germination %	Zn sulfate increased grain Zn by ~29%; line ERF 85–15 showed highest accumulation	Genetic variability exists for Zn absorption; Zn improves seed vigor. Polishing causes 28% loss.
Agronomic biofortification	Abbas et al. (2009)	Wheat	Pakistan	Field experiment Alkaline, Zn-responsive soil	Zn up to 8 kg ha^-1^; higher rates also tested	Soil application	Total Zn, Mn and Fe uptake	Zn rates up to 8 kg ha^-1^ increased total Zn and Mn uptake and enhanced Fe uptake; higher Zn rates reduced Fe uptake.	Shows dose-dependent synergy/antagonism between Zn and Fe; excessive Zn can impair Fe nutrition.
Agronomic biofortification	Sánchez-Rodríguez et al. (2017)	Wheat	Spain	Field experiment Calcareous Vertisols. Zn-DTPA 0.20–0.58 mg kg^-1^ and Olsen P 2.6–31.3 mg kg^-1^	50 mg P kg^-1^ and/or 2 mg Zn kg^-1^	Soil application	Grain Zn and P/Zn ratio	Grain Zn was negatively correlated with Olsen P (R²=0.501; P<0.001). P reduced grain Zn from 20.4 to 12.0 mg kg^-1^; Zn addition increased it to 36.8 mg kg^-1^. P increased the P/Zn ratio from 125 to 279.	Demonstrates strong P antagonism in calcareous soils; soil diagnosis is essential before Zn fertilization.
Agronomic biofortification	Ahammed et al. (2025)	Potato genotypes	Bangladesh	Field experiment Silty-loam soil	Zn soil: 8 kg ha^-1^ Zn foliar: 4 kg ha^-1^	Zn soil at planting. Zn foliar at 45 and 60 days after planting	Tuber Zn concentration, total Zn uptake, yield	Mean tuber Zn uptake and yield were positively correlated, and both depended on genotype, dose and method. Foliar applications were often more efficient than soil application.	Recommends reporting total Zn uptake as well as concentration because high concentration may coincide with low yield.
Agronomic biofortification	Cipriano et al. (2025)	Sorghum (*Sorghum bicolor*)	Brazil	Field experiment	Sodium selenate (20, 40, 80 g ha^-1^), K-hydroxy-selenide, and acetylselenide	Foliar spray twice (flowering and grain-filling)	Yield, protein, Se content/uptake, macro- and micronutrients	Sodium selenate significantly increased grain Se; 80 g ha^-1^ was the most effective dose	Sodium selenate is the best source for sorghum. Site-specific calibration is crucial. Single-season study.
Agronomic biofortification	Xue et al. (2023)	Maize cultivars ZD958, DH605 and LD510	China	Field experiment Multi-nutrient biofortification	Urea, ZnO nanoparticles, chitosan-Zn nanoparticles, ZnSO_4_·7H_2_O, and cocktail of urea+ZnSO_4_·7H_2_O+FeSO_4_·7H_2_O+Na_2_SeO_3_	Three applications from tasseling stage	Grain Zn, Fe, Se, N, yield, phytic acid, estimated absorption	Grain Zn ranged 11.2–31.8 mg kg^-1^; increases were 36.6–134.0% (ZD958), 11.1–77.1% (DH605) and 18.5–74.9% (LD510). Cocktail increased Fe up to 29.4%, Se from 21.4 to 413.5 µg kg^-1^ and N by 4.4–7.1%, without major yield effects.	Nanofertilizers did not consistently outperform ZnSO_4_. Cultivar, formulation, dose and environment determined efficiency.
Agronomic biofortification	Ierna et al. (2020)	Potato cv. Bellini	Italy	Field experiment Mildly alkaline soil: pH 7.5, CaCO_3_ 3.3% and Zn 2.7 mg kg^-1^	Multi-micronutrient foliar fertilizer with B, Cu, Fe, Mn, Mo and Zn	Three applications during the growing season	Tuber Zn and Fe, yield/quality	Tuber Zn increased by >26% and Fe by 68% at harvest.	Supports multi-micronutrient foliar enrichment of tubers under mildly alkaline conditions
Agronomic biofortification	Namorato et al. (2025)	Common Bean	Brazil	Greenhouse (Pot study)	Se-enriched urea and ammonium sulfate (0.94 mg pot^-1^)	Soil top-dressing applied 20 days after emergence	Seed yield, Se/S concentration, gas exchange, centesimal composition	Se-urea was more effective than ammonium sulfate; max Se reached 11.17 µg g ^-1^	Se-urea increases availability via localized pH increase. Genotypic responses vary significantly.
Agronomic biofortification	Zou et al. (2019)	Wheat cultivars	China, India, Mexico, Pakistan, South Africa, Turkey	Multi-country field trials	Cocktail: 0.5% ZnSO_4_·7H_2_O; 0.04% KIO_3_; 0.001% Na_2_SeO_4_; 0.2% Fe-EDTA	Foliar application twice	Grain Zn, I, Se and Fe	Zn increased from 28.6 to 46.0 mg kg^-1^ with Zn alone and to 47.1 mg kg^-1^ with the cocktail. Selenate effectively increased grain Se in India and Pakistan.	Demonstrates feasibility of multi-nutrient foliar biofortification across environments.
Agronomic biofortification	Quispe et al. (2025)	Broccoli	Brazil	Greenhouse (Pot study)	Se (multinutrient fertilizer), SeOrg, and Na_2_SeO_4_ (Foliar: 25–50 g ha^-1^; Soil: 50–100 g ha^-1^)	Seedling priming + Soil or Foliar adult phase application	Head production, Se content, post-harvest processing losses	Seedling priming + foliar Se (multinutrient fertilizer) maximized floret Se accumulation	Priming maximizes adult absorption. Foliar is more effective than soil. Risk of production loss at high Se.
Agronomic biofortification	Morais et al. (2025)	Soybean	Brazil	Field experiment pH(CaCl_2_) = 4.9	Zinc sulfate (ZnSO_4_·7H_2_O), 2 kg ha^-1^ (sowing) + foliar supplementary application	Foliar spray at reproductive stages R3 and R5	Chlorophyll, leaf/grain Zn content, productivity	Zn at R3+R5 increased productivity (by 318 kg ha^-1^) and grain Zn (reaching 37.7 mg kg^-1^)	Splitting fertilizer applications during the reproductive stage helps reduce nutrient losses. However, the results were constrained by climatic factors and disease.
Agronomic biofortification	Kromann et al. (2017)	Potato cvs. INIAP-Natividad and Chaucha roja	Ecuador	Field experiment soil pH: 6.29 Andisols	Zn soil: 10, 20 and 40 kg ha^-1^ Zn foliar: 1.25, 2.5, 5 and 10 kg ha^-1^ Fe Soil: 40 kg ha^-1^ Fe foliar: 6.71 mM	Soil-applied Zn and Fe at planting. Foliar Zn and Fe applied five times at weekly intervals, beginning approximately 55–60 days after planting.	Tuber Zn and Fe Yield	Combined treatment increased tuber Zn up to 2.69-fold without affecting yield. In field, foliar application increased Zn 2.51-fold and soil application 1.91-fold in INIAP-Natividad.	Zn Foliar application was generally more efficient, while soil fertilization remained useful at lower pH. Fe fertilization did not consistently increase tuber Fe
Genetic and biotechnological biofortification	Upadhayay et al. (2022)	Rice germplasm (diverse genotypes)	India	Field experiment Multi-genotype and multi-environment evaluation	No fertilizer treatment	Germplasm screening	Grain Zn and Fe, agronomic traits and heritability	Substantial variation was detected among genotypes, with moderate-to-high heritability for several mineral and agronomic traits, indicating that a meaningful proportion of the phenotypic variation can be exploited through selection.	Supports conventional breeding as a feasible route for improving grain mineral density while retaining agronomic adaptation; validation across environments remains essential.
Genetic and biotechnological biofortification	Senguttuvel et al. (2023)	Rice germplasm (diverse genotypes)	India	Postharvest comparison of brown and polished rice	No fertilizer treatment	Germplasm screening	Zn and Fe concentrations in brown and polished grain	Zn ranged from 6.2 to 71.6 mg kg^-1^ in brown rice and from 4.8 to 40.9 mg kg^-1^ in polished rice. Iron ranged from 6.9 to 67.3 mg kg^-1^ in brown rice and from 0.8 to 36.45 mg kg^-1^ in polished rice.	Confirms broad exploitable genetic variation but also demonstrates that polishing can markedly reduce the mineral content delivered to consumers.
Genetic and biotechnological biofortification	Tong et al. (2022)	Wheat (166 cultivars)	China	Four contrasting environments	No fertilizer treatment	Genomic association analysis	Grain Zn, Fe, heritability, loci/QTL	Zn ranged 21.8–58.9 mg kg^-1^ and Fe 32.5–74.6 mg kg^-1^; heritability was 0.71 for Zn and 0.72 for Fe. Forty-one loci were identified (25 Zn, 16 Fe), including 17 putative novel Zn QTL and 8 novel Fe QTL.	Provides markers and loci for breeding; validation across backgrounds and environments remains necessary.
Genetic and biotechnological biofortification	Pypka et al. (2025)	Tobacco (*Nicotiana tabacum*)	Poland	Controlled heterogeneous +Zn/−Zn root supply	14-day heterogeneous +Zn/−Zn root treatments	Controlled growth experiment	Expression of *NtZIP4B*, *NtHMA4a/b*, *NtNAS*; root status profiles	The −Zn NtZIP4B profile dominated in 70% of deficient roots, whereas the sufficiency profile dominated in 90% of +Zn roots, indicating systemic coordination of import, export and nicotianamine chelation.	Mechanistic evidence rather than direct food biofortification; supports target-gene selection for future engineering.
Genetic and biotechnological biofortification	Hu et al. (2023)	Rice	China	Genetic characterization of leaf senescence and node-I transport	Manipulation/characterization of OsHMA2; no fertilizer dose	Leaf senescence and node-I transport stages	Zn remobilization to grain, nodal retention, Cd co-transport	OsHMA2 was strongly induced in leaves and node-I and promoted Zn movement toward reproductive organs, but substantial Zn remained trapped in node-I. Improved Zn delivery occurred without increasing Cd accumulation.	Node-I is a key control point; enhancing leaf remobilization alone is insufficient.
Genetic and biotechnological biofortification	Agrawal et al. (2024)	Potato: cultivated material, native germplasm and breeding populations	Global	Systematic literature review	No fertilizer treatment; genetic and biotechnological interventions	Trait discovery, crossing, selection and gene manipulation	Fe and Zn concentration, bioavailability, candidate genes and breeding tools	The review identified multiple complementary genetic routes for increasing micronutrient uptake, transport, storage and concentration in potato tubers, including conventional crossing and targeted molecular modification.	Potato ploidy, high heterozygosity, regulatory requirements and genotype-by-environment effects complicate the development and deployment of nutritionally improved cultivars.

### 1.5 Evidence synthesis

Given the substantial heterogeneity among studies in terms of species, soil properties, experimental environments, fertilizer sources, nutrient doses, molecular approaches, and reported responses, the evidence was synthesized qualitatively rather than through meta-analysis. The synthesis focused on identifying common and nutrient-specific mechanisms, similarities and differences among Zn, Fe, Se, and I, and potential interactions across the soil–plant continuum. Particular attention was given to how crop type, soil conditions, environmental context, and management practices influence these mechanisms and responses. The synthesis also considered knowledge gaps, methodological challenges, and opportunities to translate mechanistic understanding into effective crop biofortification strategies.

## 2 Introduction

### 2.1 The global challenge of “hidden hunger” and food security towards 2050

By 2050, the projected population will exceed 9 billion people, which will exert unprecedented pressure not only on food production but also on the nutritional quality of the diet (Gharibzahedi and Jafari, 2017). It is estimated that approximately 582 million people will be chronically undernourished, while nutrient deficiency affects around 6.8% of children under 5 years of age, equivalent to 45 million individuals (Pypka et al., 2025). Likewise, hidden hunger affected between 702 and 828 million people in 2021, according to the World Health Organization (WHO) (FAO et al., 2022). Overall, micronutrient deficiency affects more than 2 billion people worldwide, constituting a serious public health problem (Abdu et al., 2022; Agrawal et al., 2024; Hacisalihoglu, 2020).

Among these deficiencies, zinc (Zn), iron (Fe), iodine (I), and selenium (Se) represent the most critical micronutrients, affecting a significant proportion of the global population. It is estimated that Zn and Fe deficiencies affect approximately 17% and 30% of the population, respectively (de Benoist et al., 2008b; Bailey et al., 2015) while iodine and selenium deficiencies impact around 30% and 14%, respectively (de Benoist et al., 2008a; Jones et al., 2017). These deficiencies have severe consequences, including impaired immune function, delayed cognitive development, and high mortality rates, particularly among vulnerable populations. One study estimated that approximately 500,000 children die due to Zn nutritional deficiency in Latin America, Africa and Asia in 2004 (Fischer Walker and Black, 2007).

Zinc deficiency constitutes a widespread and frequently underestimated nutritional problem, acting as a silent epidemic that affects 17% of the global population (Jalal et al., 2024). This deficiency is considered the fifth leading cause of disease in developing countries (WHO, 2002). Zinc is essential for multiple physiological processes in humans, acting as a cofactor for more than 300 enzymes in the body and playing an important role in cellular metabolism, immune function, growth, and development (Davaran Hagh et al., 2016; Zaman et al., 2017). Nevertheless, its deficiency is associated with severe disorders such as growth retardation, visual disorders, alterations in the development of reproductive organs, and even chronic diseases such as cancer (Ma et al., 2023). On the other hand, Fe deficiency affects 20–25% of the global population; I deficiency affects nearly 2 billion people; and Se deficiency affects between 0.5 and 1.0 billion people (Schiavon et al., 2020; Koç and Karayiğit, 2021).

In addition to Zn, Fe, I, and Se are essential elements for human health, as they participate in key functions such as oxygen transport, immune response, hormonal regulation, and antioxidant activity. Iron is essential for hemoglobin synthesis and oxygen transport, with approximate requirements of 7–10 mg day^-1^ in children, 11–15 mg day^-1^ in adolescents, and 8–18 mg day^-1^ in adults. Zinc is recommended in amounts of 11 mg day^-1^ (Institute of Medicine, 2001). Iodine, required for thyroid hormone regulation, has a recommended intake of 90 µg day^-1^ in children and 150 µg day^-1^ in adolescents and adults (Institute of Medicine, 2001), while Se, which is important for the defense of antioxidant systems, is recommended at around 55–70 µg day^-1^ in adults (EFSA Panel on Dietetic Products, Nutrition and Allergies [EFSA], 2014). These requirements vary according to the stage of life and are essential for maintaining key physiological functions of humans (Awuchi et al., 2020; Ofori et al., 2022).

The nutritional problem also has an edaphic and agricultural basis. Zinc deficiency in soils may affect up to 50% of the world’s agricultural land, and more than 49% of arable land exhibits conditions that favor its low availability, including calcareous soils, intensively cultivated soils, sandy soils, or soils with high phosphate fertilization (Hacisalihoglu, 2020; Pypka et al., 2025). In rice, Zaman et al. (2017) reported that more than 30% of the world’s soils are deficient in Zn. In this context, hidden hunger also depends on the capacity of agricultural systems, including soils, to supply essential elements.

### 2.2 The core challenge: balancing agricultural yield with nutritional quality in crop production

The central challenge of modern agricultural production is not only to increase yield but also to balance it with nutritional quality. For decades, breeding programs and agronomic management systems have been primarily focused on increasing the production of staple foods; however, this strategy has not always ensured higher micronutrient concentrations. As a result, populations that depend on diets based on cereals, roots, and tubers may achieve sufficient caloric intake while still being exposed to deficiencies of Zn, Fe, I, and Se.

According to Koç and Karayiğit (2021), over 820 million individuals are facing hunger, while 2 billion people suffer from micronutrient shortages. Additionally, 2 million people are either overweight or obese due to these reasons, thus pointing out a burden of triple malnutrition. Processed wheat and rice lose most of their nutritional value during processing, with rice polishing taking away about 80% of thiamine. Furthermore, wheat’s milling process takes away important fractions of zinc and iron from the grain.

The effects of climate change make it even more difficult to maintain the equilibrium between yield and quality of nutrition. Under the 2050 projections for CO_2_ concentrations of up to 550 mg kg^-1^, the number of people affected by Zn deficiency may rise by 175 million, while the number of children with Fe deficiency could reach 311 million and that of people with insufficient protein intake may account for 122 million (Smith and Myers, 2018). This means that agricultural systems of the future must keep the existing level of yield.

Biofortification emerges as a response to this dilemma, as it seeks to increase the concentration or bioavailability of micronutrients in commonly consumed crops without modifying dietary patterns (FAO et al., 2022). Evidence from human studies supports its potential: provitamin A-biofortified maize (predominantly β-carotene) increased body stores of vitamin A in children in Zambia, demonstrating that this crop is an effective source and can contribute to reducing deficiency in this micronutrient (Gannon et al., 2014). Similarly, provitamin A biofortified orange-fleshed sweet potato significantly increased vitamin A intake in Mozambique (Hotz et al., 2012a) and Uganda (Hotz et al., 2012b). Furthermore, promoting the consumption of biofortified orange-fleshed sweet potato in Mozambique was associated with improved child health outcomes, including a lower prevalence of diarrhea (Jones and de Brauw, 2015). Finally, the consumption of iron biofortified beans over a period of 128 days increased hemoglobin concentration by 4.2 g L^-1^ in Rwandan women (Haas et al., 2016). However, for biofortification to be effective, programs must consider bioavailability, acceptability, costs, target population, final product quality, and sufficient consumption of biofortified food (Bhutta et al., 2013).

Therefore, the challenge is to produce foods capable of providing bioavailable micronutrients at levels that are relevant to human health. This requires integrating agronomic interventions, genetic improvement, biotechnology, human nutrition, adoption policies, and impact assessment in vulnerable populations. Biofortification should be understood as a complementary strategy to dietary diversification, supplementation, and industrial fortification, particularly in regions where diets rely on staple crops and access to diverse foods remains limited (Sharma et al., 2020; Younas et al., 2022).

### 2.3 Scope and contribution of this review within the framework of the future of biofortification

This review is framed within the concept of “the future of biofortification” understood as food systems oriented not only toward increasing production but also toward improving the nutritional density, bioavailability, and safety of micronutrients in staple crops.

In this context, the scope of this review encompasses the soil–plant interactions that regulate the mobility, acquisition, transport, and accumulation of Zn, Fe, Se, and I, and how these processes can be leveraged to design biofortification strategies. Specifically, it covers the continuum from soil availability of micronutrients and chemical speciation to uptake by roots or aerial tissues, translocation through the xylem and phloem, accumulation in edible organs, and ultimately dietary bioavailability and contribution to human nutrition (Sharma et al., 2020; Younas et al., 2022).

The review addresses the main approaches to biofortification, *i.e.*, agronomic, genetic, and biotechnological biofortification, and analyzes recent proposals aimed at their integration. Building on the foundational biofortification framework of White and Broadley (2005), agronomic biofortification includes soil applications, foliar applications, nanofertilizers, biofertilizers, and multi-nutrient cocktails (Ramzan et al., 2020). Genetic and biotechnological biofortification considers the selection of genotypes, conventional breeding, molecular markers, QTLs, gene editing, and the manipulation of transporters and genes associated with mineral homeostasis, including ZIP, IRT1, YSL, FRO2, and SULTR family members (Sharma et al., 2020; Kumari et al., 2022). Finally, integrated approaches combine agronomic management, genetic potential, soil diagnosis, bioavailability, and social acceptance (Matumba et al., 2025).

In this regard, Senguttuvel et al. (2023) mention HarvestPlus through the Biofortification Priority Index, which prioritized Zn biofortification in Asian countries such as Bangladesh, Laos, Cambodia, Myanmar, Indonesia, Sri Lanka, Nepal, Vietnam, Thailand, and India, and in African countries such as Sierra Leone, Guinea, Madagascar, Guinea-Bissau, Liberia, Mali, Comoros, Gambia, Côte d’Ivoire, and Tanzania. This demonstrates that the impact of biofortification depends on directing interventions toward regions where dependence on staple crops, micronutrient deficiencies, and vulnerable populations coincide. These interacting dimensions are integrated in the conceptual framework presented in **Figure 2**, which links soil properties, rhizosphere processes, plant acquisition and transport mechanisms, and biofortification strategies with their implications for human nutrition.

**Figure 2:**
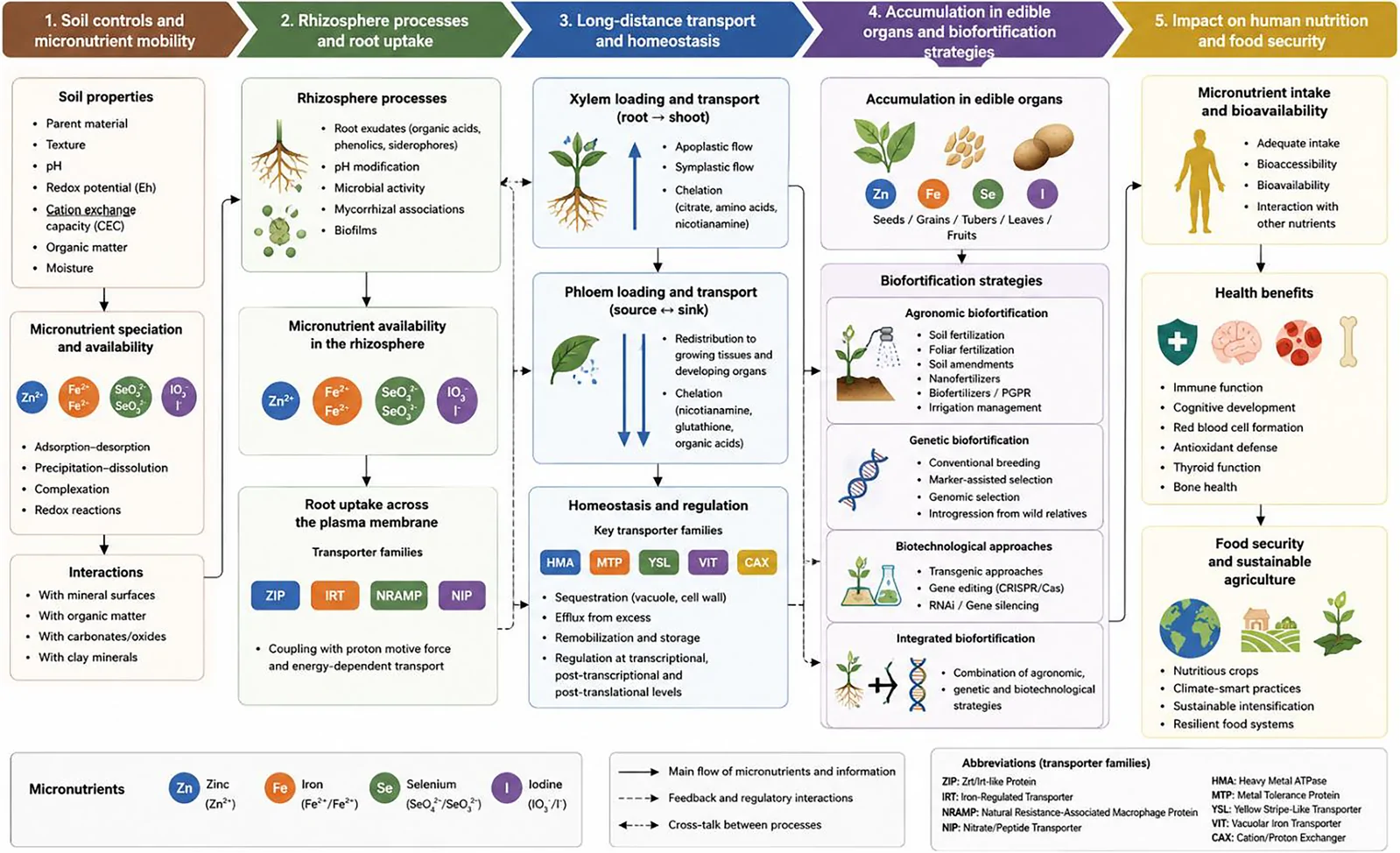
Conceptual framework of soil–plant continuum regulating the mobility and acquisition of Zn, Fe, Se, and I and their implications for crop biofortification strategies and human nutrition.

## 3 Soil mechanisms governing the mobility and availability of Zn, Fe, Se, and I

The solubility of micronutrients is strongly conditioned by soil physicochemical properties, which determine the complex interaction within the soil–plant system regarding the uptake capacity and availability of elements (Alloway, 2008). Among these determining factors are parent material (rock, originating minerals), geochemical and pedogenic processes, soil pH, soil texture, cation exchange capacity (CEC), redox potential, and interactions between nutrients and fertilizers (Jalal et al., 2024; Schiavon et al., 2020; Taşpınar et al., 2025; Moreno-Lora and Delgado, 2020), which play a determining role in the dynamics of elements such as zinc, iron, iodine, and selenium (Alloway, 2008; Kabata-Pendias and Pendias, 2001; Jalal et al., 2024; Schiavon et al., 2020).

### 3.1 Influence of soil parent material, texture, mineralogy, and cation exchange capacity

Soil parent material and its mineralogy determine the geochemical composition and soil properties. Developed soils derived from shales, schists, and basalts retain more micronutrients due to their adsorption capacity and high specific surface area; these soils may be enriched in As, Se, Cd, Cu, Mo, and Zn, although this depends on other factors (Alloway, 2008; Schiavon et al., 2020). Clay soils involve a negatively charged colloidal system where positively charged nutrients are attracted to the surface of the diffuse double layer due to the Cation Exchange Capacity (CEC), compared with sandy soils, which have low CEC (Hafeez et al., 2013; Praharaj et al., 2021; Taşpınar et al., 2025). Sandy soils derived from sedimentary rocks such as sandstones or quartz deposits are associated with deficiencies of B, Cl, Cu, Fe, Mn, Mo, Ni, and Zn due to their low clay content and reduced CEC (Alloway, 2008).

For Zn, Gong et al. (2020) reported that silty soils retain more Zn than sandy loam and organic soils, which exhibit greater deficiency of this element. In soils, Zn fractions are distributed in the water-soluble pool in the soil solution, exchangeable pool bound to particles by electrical charges, organically bound pool (adsorbed, chelated or complexed), the pool of zinc sorbed non-exchangeable onto clay minerals and insoluble metallic oxides, and the pool consisting of primary minerals undergoing weathering (Alloway, 2008).

In tropical systems such as the eastern plains of Colombia and Venezuela and the Brazilian Cerrado, Zn was reported as a deficient micronutrient (Alloway, 2008). Taşpınar et al. (2025), in their study of semi-arid agricultural lands in Eskişehir and Kütahya, Turkey (50.31 ± 23.03 mg total Zn kg^-1^), reinforce that Zn concentration is related to the chemical composition and weathering of the parent material. Other soils, such as those derived from gneiss, granite, sandstones, and limestone, tend to have low total Zn; for example, quartz in sandy soils contains low concentrations of 1–8 mg Zn kg^-1^ (Hafeez et al., 2013). Zinc in soils is found associated with minerals such as sphalerite (ZnS), franklinite (ZnFe_2_O_4_), Zn-bearing magnetite, willemite, hemimorphite, and zincite (Robe et al., 2025). de Francisco et al. (2024) compared two soils in Spain – an acidic Luvisol with sandy materials (pH = 5.9; sandy texture) and a calcareous Fluvisol (pH = 8.2; silty texture) developed on alluvial deposits – and showed that the DTPA-extractable Zn content was lower in the Luvisol due to the presence of quartz-rich parent materials with low specific surface area and reduced CEC compared with the Fluvisol, which contains more silt, mineral colloids, and higher organic matter content that retain more Zn. According to Oliveira et al. (2024), in a study conducted in Brazil in Nova Andradina, the Typic Quartzipsamment soil with sandy texture (pH = 4.8) showed low DTPA-extractable Zn, with 0.45 mg dm^-3^. Other soils such as Calcisols, Rendzinas, Leptosols, Vertisols, calcareous soils such as Calcisols, and saline soils such as Solonchaks and Solonetz present Fe and Zn deficiencies due to extensive carbonate precipitation (Alloway, 2008).

For Se, a study conducted by Dinh et al. (2018) in soils from Hainan, China, found that clay minerals reduce Se solubility because it is adsorbed as oxyanions, as observed in fractions <0.025 m compared with particles >1 mm. In the Earth’s crust, Se content ranges between 0.05–0.09 mg kg^-1^ (Neal and Sposito, 1989), while the global average is 0.4 mg kg^-1^ (Fordyce, 2007). Tropical soils may contain between 2–4.5 mg kg^-1^, and seleniferous soils between 0.5–1200 mg Se kg^-1^ (Fernández-Martínez and Charlet, 2009; Schiavon et al., 2020). Soils derived from igneous rocks such as granite and other volcanic materials are poor in Se, whereas sedimentary rocks such as shales are rich in Se (Schiavon et al., 2020). Field evidence from the Mancos Shale in Colorado showed that irrigation mobilized soluble Se-bearing salts and gypsum, while a substantial fraction of Se in irrigated soils became associated with organic matter (Mast et al., 2014). The direction and mechanistic basis of the main edaphic effects are summarized in **Table 1**.

### 3.2 Soil pH and redox potential as master regulators of micronutrient speciation

The distribution of an element in soil is determined by a dominant factor known as pH, which influences the solubility and speciation of elements. An increase of pH unit can decrease Zn^2+^, Fe^2+^ and Fe^3+^ availability (Senguttuvel et al., 2023). In the soil solution, the zinc concentration is approximately 10–^4^ M (6.5 mg kg^-1^) at pH 5.0 and 10–^10^ M (0.007 mg kg^-1^) at pH 8.0 (Lindsay, 1991). Under alkaline conditions (pH > 7), Zn is predominantly found in less available forms such as Zn(OH)^+^ (pH > 7.7) or forming insoluble compounds such as ZnCO_3_ and Zn(OH)_2_ (pH > 9.1), thereby reducing Zn solubility and plant uptake (Alloway, 2008; Kaur et al., 2024; Silva-Herrera et al., 2025; White and Broadley, 2011).

Selenium chemical speciation is largely influenced by soil redox potential and pH, showing general tendencies rather than universal rules. In oxidizing and alkaline environments, selenate (SeO_4_^2-^) typically tends to be a prevalent inorganic form in the soil solution due to its high mobility and low adsorption. Conversely, under acidic and moderately reducing conditions, selenite (SeO_3_^2-^) is frequently found as a dominant species, though its actual availability is further moderated by strong adsorption onto Fe and Al oxides and complex interactions with soil organic and microbial activity. In reducing environments, Se may occur as selenide (Se^2-^) or H_2_Se, which are forms with very low mobility (Deng et al., 2025; Huang et al., 2026).

Iodine speciation is linked to the redox state. In oxygenated environments, the oxidation of iodide (I^-^) to molecular iodine (I_2_) and iodate (IO_3_^-^), predominates, whereas in reducing environments, the reduction of iodate (IO_3_^-^) to iodide (I^-^) occurs (Jiang et al., 2024).

#### 3.2.1 Zinc and iron solubility constraints under calcareous, alkaline and saline soils

Calcareous or alkaline soils, characterized by a high CaCO_3_ content in the parent material and a pH between 7.0 and 8.5, cover almost one-third of the world’s land surface area (Bolan et al., 2023) and represent one of the greatest limitations for Zn and Fe availability due to adsorption, precipitation, and reduced diffusion processes in the soil matrix (Schiavon et al., 2020; Hacisalihoglu, 2020; Praharaj et al., 2021; Sánchez-Palacios et al., 2023). Nearly 50% of agricultural soils used for cereal production worldwide are Zn deficient, particularly alkaline and calcareous soils, sandy soils, and soils with high phosphate fertilization (Ajeesh Krishna et al., 2020; Sadeghzadeh, 2013).

In Mexico, Calcisols occupy 10.4% of the national territory, and a 20% reduction in yield has been found in pecan orchards associated with Zn deficiency (Martínez-Ríos et al., 2024). In a soil study in India where more than 30 million samples were analyzed (pH between 7.9 and 8.3), 39% were found to be deficient in total Zn (<0.6 mg kg^-1^), mainly due to alkalinity and the presence of carbonates (Khokhar et al., 2024). Calcareous soils in rice fields in China, alkaline soils with low organic matter content in Turkey, soils from southern Austria, and soils from southwestern Australia are some examples of Zn-deficient soils worldwide (Sadeghzadeh, 2013; Saleem et al., 2020).

In a study conducted in Andalucia, southern Spain, where twenty calcareous Vertisols were analyzed, it was demonstrated that Zn concentration was <0.50 mg kg^-1^ due to high CEC, low organic carbon (OC <16 g kg^-1^), and the predominance of smectites (Sánchez-Rodríguez et al., 2017). In Mediterranean soils of Spain, DTPA-extractable Zn values ranged from 0.1 to 5.7 mg kg^-1^, where calcareous soils showed three times less Zn than non-calcareous soils, mainly due to Zn–P–Fe oxide complexes (Recena et al., 2021). In Iran (33–34% CaCO_3_, pH 7.4–7.6), total Zn was 51–55 mg kg^-1^, but available Zn (DTPA-extractable) was only 0.15–0.18 mg kg^-1^, demonstrating that even when total Zn is high, there is a strong limitation in the solubility of this element (Norouzi et al., 2014).

Under calcareous conditions, Zn^2+^ precipitates in the form of ZnCO_3_ and Zn(OH)_2_, reducing the concentration of Zn^2+^ in the soil solution (Senguttuvel et al., 2023; Taşpınar et al., 2025; Martínez-Ríos et al., 2024). Additionally, it can react with groups such as HPO_4_^2-^ and PO_4_^3-^, forming insoluble compounds (de Francisco et al., 2024).

The solubility of Fe decreases 1,000-fold for every one unit increase in soil pH, with serious availability problems at values above 7 (Koç and Karayiğit, 2021). In addition, Senguttuvel et al. (2023) observed that iron chlorosis in calcareous soils is more severe due to the low availability of Fe for plants, as it precipitates in the form of Fe(OH)_3_. In aerated and alkaline soils, Fe solubility is extremely low, with concentrations below 10–^15^ M, and it occurs in retained forms such as Fe hydroxides, oxides, and oxyhydroxides (Broadley et al., 2012). In contrast, under gleyed and flooded soils, the reduction of Fe oxides increases its solubility; however, Zn can form zinc sulfide compounds (ZnS) under increasing pH conditions in reducing environments (Alloway, 2008).

In contrast, saline soils generate osmotic stress and ionic competition that limit micronutrient uptake, particularly due to the presence of Na^+^ and Cl^-^, which interfere with the uptake of essential cations such as Zn^2+^ and Fe^2+^ (Praharaj et al., 2021; Moreno-Lora and Delgado, 2020).

Therefore, the low availability of Zn and Fe in calcareous, alkaline, and saline soils is primarily determined by soil chemical processes, which reduce the efficiency of agronomic biofortification strategies based on the application of fertilizers to the soil.

#### 3.2.2 Selenium chemical speciation (selenate vs. selenite) and bioavailability

The chemical speciation of selenium in soil directly influences its bioavailability and mobility in the soil–plant continuum (Wang et al., 2017; Fernández-Martínez and Charlet, 2009; Dinh et al., 2018). The global average Se concentration in soils has been estimated at 0.322 ± 0.002 mg Se kg^-1^ (Jones et al., 2017). These low concentrations, together with the limitations of available analytical methods, have hindered the identification and characterization of different chemical species of Se, especially organic forms (Tolu et al., 2022). This makes the complete characterization of its chemical species difficult, including organic and inorganic forms, molecular size, and electrical charge. In addition, the role of pH operates mainly indirectly, by shifting Se speciation and governing the pH-dependent adsorption of selenite onto Fe and Al oxides, whereas the contribution of soil organic matter to Se bioavailability remains less well-defined (Li et al., 2016; Deng et al., 2025). Selenium bioavailability is influenced by soil organic matter (SOM), the mineral composition of clays, Fe and Al oxides, pH, and soil microbial activity (Deng et al., 2025).

Selenium can occur in oxidation states ranging from -2 to +6. In soils with high redox potential and alkaline conditions, selenate (SeO_4_^2-^; Se(VI)) predominates, which is an inorganic soluble form that is mobile, soluble, and has low adsorption by oxides (Schiavon et al., 2020; Deng et al., 2025), with greater availability for plants (Wang et al., 2017). However, as the redox potential decreases, selenate is progressively reduced to selenite (SeO_3_^2-^; Se(IV)), a less mobile form that is strongly adsorbed by iron and aluminum oxides or clay minerals, particularly at low pH. Under these conditions, selenite can further transform into elemental Se (Se^0^) or selenides (Se^2-^), which are increasingly less soluble forms and therefore less available to plants (Schiavon et al., 2020; Fernández-Martínez and Charlet, 2009; Dinh et al., 2018; Wang et al., 2017). Selenide generally occurs as metal selenides or hydrogen selenides (H_2_Se), both of which exhibit low mobility under reducing conditions (Deng et al., 2025; Schiavon et al., 2020). Organic selenium occurs as selenoamino acids (methylselenides, selenomethionine, and selenocysteine), whereas volatile organic species include methylated compounds such as dimethyl selenide and dimethyl diselenide (Deng et al., 2025).

A significant fraction of Se is associated with SOM, although its contribution to Se bioavalability remains incompletely defined (Li et al., 2016). Although organic Se is generally considered a relatively inmobilized fraction, it may become a source of plant-available Se through dissolution, mineralization, microbial transformations, and gradual degradation of organic matter (Supriatin et al., 2016; Tolu et al., 2022; Wang et al, 2017). These processes can release soluble forms of Se, lead to oxidation of organic Se which can be transformed into selenite or selenate and possibly provide the plants with selenoamino acids directly and thus increase the Se availability in soil (Dinh et al., 2018; Tolu et al., 2022; Wang et al., 2017). Soil type also plays an important role in Se bioavailability. In the tropical soils, Se(IV) is fixed by means of a specific sorption process known as inner-sphere complexation, while the sorption capacity of Se(VI) is much higher in soils with less than 22% of clay content than the capacity of Se(IV) (Araujo et al., 2020; Deng et al., 2025).

On the other hand, due to its chemical similarity to sulfur (S), Se can substitute for S in amino acids, becoming incorporated into proteins, and other organic metabolites (Dinh et al., 2018; Tolu et al., 2022). Furthermore, its geochemical behavior resembles that of arsenic (As), which influences its adsorption, mobility and interaction with soil minerals and organic matter (Deng et al., 2025).

In general, selenium bioavailability is governed by dynamic interaction among its chemical species, a process regulated by redox conditions, soil mineralogy, organic matter and microbial activity, which ultimately determines its mobility and uptake by plants.

#### 3.2.3 Iodine complexity in the soil matrix: inorganic transformations and atmospheric inputs

Iodine (I) presents one of the most complex and least understood dynamics within the soil–plant continuum. Most of the iodine in soil originates from atmospheric sources, specifically through the volatilization of inorganic forms from seawater, which enter the soil–plant continuum through precipitation and deposition (Shetaya et al., 2012). A significant portion of atmospheric iodine is derived from iodine (I^-^), which reacts with atmospheric ozone (O_3_) to form volatile inorganic gaseous species, called hypoiodous acid (HOI) and molecular iodine (I_2_). The emissions are estimated to account for nearly 80% of atmospheric iodine; they undergo photolysis driven by solar radiation, producing atomic iodine (I) which reacts with ozone to form IO radicals that deplete tropospheric ozone (Jiang et al., 2024). Iodate (IO_3_^-^) and iodide (I^-^) are among the dominant inorganic species; their concentrations in seawater range from 0.45 to 0.50 μmol L^-1^, allowing inorganic emissions to contribute approximately 2 Tg of iodine year^-1^ at the global scale. Additionally, volatile organoiodine compounds, such as CH_3_I, CH_2_I_2_, CH_2_IBr, and CH_2_ICl, contribute approximately 0.6 Tg of iodine year^-1^ (Jiang et al., 2024; Pound et al., 2024).

The transformations of these iodine species are controlled by microbial processes that can reduce IO_3_^-^ to I^-^ under anoxic conditions using organic compounds such as acetate or lactate as electron donors. Among these species, *Denitromonas* sp. IR-12, *Pseudomonas* sp. SCT, and *Shewanella oneidensis* MR-1 have been considered, as they possess specific reductase enzymes for this process (Jiang et al., 2024). Iodine returns to the soil surface via dry and wet deposition, subsequently redistributing through groundwater flow and sediment transport (ATSDR, 2004). In soils, it occurs as iodate (IO_3_^-^), iodide (I^-^), and various forms of organic iodine (Org-I) (Steinberg et al., 2008a; Pound et al., 2024).

Its presence and chemical forms are controlled by soil factors such as pH and redox potential. Iodine in soil occurs in several oxidation states and in a variety of inorganic and organic forms, including iodide (I^-^), iodate (IO_3_^-^), elemental iodine (I_2_), and organic iodine (White et al., 2009; Yang et al., 2007; Yoshida et al., 2007; Rakoczy-Lelek et al., 2021). Dai et al. (2009) state that iodate is less mobile due to the formation of complexes between IO_3_^-^ and Fe/Al, whereas I^-^ is adsorbed through weak electrostatic interactions. Inorganic iodine forms can be retained in acidic soils with low redox potential through adsorption by iron and aluminum oxides, where iodide (I^-^) predominates (Gallard et al., 2009; Shetaya et al., 2012; Rakoczy-Lelek et al., 2021). Dai et al. (2009) and Rakoczy-Lelek et al. (2021) indicate that as pH decreases, positive charges increase in the colloidal surface, favoring the adsorption of anions such as iodide (I^-^). Under these conditions, iodide (I^-^) can be lost through oxidation and reaction with iodate according to: IO_3_^-^ + 5I^-^ + 6H^+^ → 3I_2_ + 3H_2_O, promoting the formation of molecular I_2_ (Yang et al., 2007). Meanwhile, iodate (IO_3_^-^) can be adsorbed even up to neutral to alkaline pH and under oxidizing conditions (Rakoczy-Lelek et al., 2021).

When comparing soil iodine forms—such as IO_3_^-^ and I^—^in terms of plant availability, it has been found that although IO_3_^-^ can be directly absorbed by the plant (Riyazuddin et al., 2023), it is reduced to I^-^ before it can be effectively assimilated (Dobosy et al., 2024). This reduction occurs primarily in the roots through the activity of iodate/nitrate reductase enzymes (Medrano-Macías et al., 2016). Similarly, the availability of soil I_2_ is severely limited by its rapid covalent binding to organic matter, although it remains a significant source for foliar uptake from the atmosphere (Medrano-Macías et al., 2016).

Inorganic iodine can be rapidly transformed into organic forms through reduction processes and reactions with organic compounds when it forms stable bonds, mainly with aromatic structures (Fukui et al., 1996; Schlegel et al., 2006). Iodine dynamics in soil are strongly dominated by its interaction with SOM, with humus being the main iodine reservoir in soils (Dai et al., 2009; Steinberg et al., 2008b; Rakoczy-Lelek et al., 2021; White and Broadley, 2005). In addition, transformations such as the oxidation of iodide to reactive intermediates (I_2_, HOI) facilitate its incorporation into SOM, especially under low pH conditions (Bichsel and von Gunten, 1999; Warner et al., 2000).

### 3.3 The dual role of soil organic matter and humus in micronutrient retention and complexation

Soils with more than 10% organic matter, such as peatlands, are associated with Cu, Mn, and Zn deficiencies. These soils include Histosols, Podzols, Rendzinas, Umbrisols, Cambisols, Fluvisols, and Gleysols (Alloway, 2008). Humic acids, the main fraction of SOM, have an adsorption capacity ranging from 300–1400 meq 100 g^-1^, which exceeds the adsorption capacity of other mineral fractions by 2- to 30-fold; this controls mobility, speciation, chelation, and other processes (Boguta and Sokolowska, 2016).

Among other edaphic processes that condition Zn mobilization are: (1) The addition of organic matter and microbial activity, which influence Zn availability through processes such as biosorption, bioaccumulation, and bioprecipitation (Jain et al., 2020), since organic decomposition and root exudates produce organic acids capable of forming stable soluble complexes (Sánchez-Palacios et al., 2023; Luo et al., 2008). However, kinetic processes such as Zn fixation or release can reduce its availability over time (Maqsood et al., 2011; Donner et al., 2010). Therefore, organic matter plays a dual role by reducing solubility through adsorption or increasing it through the complexation of soluble compounds (Jalal et al., 2024); (2) Interactions with phosphorus can limit its uptake and translocation in plants (Sánchez-Rodríguez et al., 2017).

In a soil study from Poland (pH 4.45–7.88, organic C between 0.22–3.48%, CEC between 2.51–45.6 cmol kg^-1^), where humic acids were isolated, it was observed that soil type and CEC condition the Zn–humus interaction (Boguta and Sokolowska, 2016). On the one hand, SOM can increase Zn solubility through complexes with organic acids, while it can also reduce it through adsorption (Boguta and Sokolowska, 2016; Norouzi et al., 2014). In the case of Zn, at pH values higher than 7, complexes between Zn and SOM increase due to the dissociation of carboxylic acid groups (-COOH), carboxylates (-COO^-^), and hydroxyl groups (-OH), as well as the reduction of proton competition or deprotonation (H^+^) for adsorption sites (Boguta and Sokolowska, 2016). Root exudates, low-molecular-weight organic compounds released by plant roots, also form stable complexes with Zn, allowing an increase in its solubility (Norouzi et al., 2014).

In Iran, a soil study found that SOM increased up to 0.60% with the incorporation of clover and 0.38% under a fallow system with Sunflower (*Helianthus annuus* L. cv. Allstar), Sorghum (*Sorghum bicolor* L. cv. Speed Feed), Clover (*Trifolium pratense* L.), and Safflower (*Carthamus tinctorius* L. cv. Koseh-e-Isfahan), enhanced Zn association with the organic fraction and Zn uptake by wheat from 19.2% to 51.8% (Norouzi et al., 2014). In a soil study in Turkey, organic matter (1.7% on average) showed a positive correlation with Zn (p < 0.01) (Taşpınar et al., 2025). However, in soils from India (0.40% OM) and Pakistan (1.08% OM) with low organic matter, Zn complexation may be limited (Ram et al., 2024). Therefore, the rhizosphere–microbe–root interaction determines Zn concentration (Sánchez-Palacios et al., 2023). For iron, SOM plays a decisive role in maintaining solubility: microbial siderophores and root-derived organic acids chelate Fe(III) and keep it in solution, particularly in calcareous soils where inorganic Fe would otherwise precipitate, while humic substances also form stable Fe–organic complexes that regulate its retention (Colombo et al., 2014).

For iodine, studies in soils from China indicate that iodide (I^-^) adsorption is correlated with SOM and CEC (Dai et al., 2020). Humic substances can increase iodine adsorption, reducing its mobility in soil (Naeem et al., 2026). In many terrestrial environments, SOM–I complexation processes mediated by microorganisms occur (Jiang et al., 2024; Riyazuddin et al., 2023). In the case of Se, the incorporation of plant residues promotes the transformation of low-molecular-weight organic Se into high-molecular-weight forms, which is associated with more stable SOM–Se forms that ultimately result in more stable but less soluble Se pools (Huang et al., 2026). Among the organic forms of Se, selenomethionine and selenocysteine constitute an important fraction of bioavailable Se (Deng et al., 2025).

### 3.4 Nutrient-nutrient interactions and fertilizer management

Nutrient interactions can be synergistic or antagonistic, depending on soil properties and the chemical and biological interactions present (Senguttuvel et al., 2023). Das and Green (2016) reported antagonistic interactions with P, S, Ca, and Mg, as well as with other micronutrients such as Fe, Mn, Cu, and Mo. These interactions influence nutrient solubility, adsorption, transport, and plant uptake.

Among these interactions, phosphorus and organic matter might affect the spatial variability of nutrients (Taşpınar et al., 2025). On the other hand, fertilizer type, application rate, and nutrient balance strongly influence micronutrient solubility and availability. Hacisalihoglu (2020) found that nearly 50% of intensively cultivated soils present Zn deficiency. Also, Pelegrino et al. (2026) have found that to accurately assess soil zinc deficiency and calculate fertilizer needs, soil properties such as pH, texture, and phosphorus levels must be integrated alongside extractable zinc, as doing so revealed that 78.3% of the Brazilian Cerrado faces a medium deficiency risk and increased estimated zinc fertilizer demand in high-risk areas. In calcareous soils under a rice–wheat cropping system, Zn was mainly associated with relatively stable soil fractions, with 78–83% occurring as residual Zn, followed by crystalline oxide-bound Zn (8.06–9.89%) and organically bound Zn (5.48–7.94%). Only a small proportion (0.38–0.87%) was present in water-soluble and exchangeable forms, reflecting the limited mobility and availability of Zn in these soils (Lakshmi et al., 2021).

In crops such as rice under flooded systems, it was found that an increase in rhizosphere pH, which allowed NO_3_^-^ uptake and OH^-^ release, restricted Zn solubility and consequently grain yield (Senguttuvel et al., 2023; Midya et al., 2021). Hamzah Saleem et al. (2022) mentioned that soils with low redox potential, high Na, high P, and cation imbalances such as a high Mg/Ca ratio decrease Zn and Fe concentrations in solution. This occurs because Zn and Fe can precipitate in the soil matrix together with P, forming insoluble compounds (Sánchez-Rodríguez et al., 2017; Silva-Herrera et al., 2025). On the other hand, due to the chemical nature of anions, selenite can compete with phosphate for adsorption sites on colloids, affecting root uptake (Fu et al., 2025).

## 4 Plant acquisition strategies: rhizosphere modifications and molecular mechanisms

Micronutrient acquisition by plants is a process that depends on nutrient solubility in the soil, the ability of roots to modify the rhizosphere, and the activation of mechanisms that enable uptake, transport, and internal homeostasis. In the case of Zn, this element is mainly absorbed as Zn^2+^ from the soil solution; however, due to its low mobility and adsorption with colloids, carbonates, phosphates, and metal oxides such as Fe and Al, plants must activate mobilization strategies that include modifying their rhizosphere (Hacisalihoglu and Kochian, 2003; Kumar et al., 2025). These strategies include proton release, organic acid exudation, and the expression of transporters (Hamzah Saleem et al., 2022). In addition, Zn is associated with the homeostasis of other nutrients such as Fe, given the use of similar transporters and uptake strategies (Römheld and Marschner, 1986; López-Arredondo et al., 2013; Senguttuvel et al., 2023). Similarly, Se use strategies associated with sulfate anion transporters (Duborska et al., 2022; Schiavon et al., 2016; Terry et al., 2000) and I use chloride channels or other anion transport systems (De Angeli et al., 2006; Rakoczy-Lelek et al., 2021; Kiferle et al., 2021; White et al., 2009). The principal chemical forms, uptake systems and intracellular fates of the four micronutrients are compared in **Table 2**.

### 4.1 Alterations of the rhizosphere for greater nutrient solubilization and uptake (acidification, proton pumps, and organic acids)

The rhizosphere constitutes a highly dynamic zone where roots release root exudates (Molnár et al., 2023). Rhizosphere activity is controlled by a complex of low- and high-molecular-weight compounds, including carbohydrates, amino acids, protons, enzymes, phytosiderophores, and organic acids capable of solubilizing nutrients (Molnár et al., 2023). In the rhizosphere, Zn availability does not depend on total Zn but rather on the soluble fraction (Hamzah Saleem et al., 2022). While Zn mobility is low in tropical regions, in areas with the presence of carbonates, phosphorus, clays, and Fe and Al oxides, this soluble fraction is reduced and, therefore, Zn availability for root uptake is also decreased (Jalal et al., 2024). Zinc reaches the root in the rhizosphere through three main processes: mass flow, when soluble Zn is high; diffusion, which dominates when solubility is low; and root interception (Kumar et al., 2016).

Meanwhile, roots can modify their rhizosphere to improve Zn acquisition by changing root architecture, increasing soil exploration, producing phytosiderophores, and releasing organic anions (Hamzah Saleem et al., 2022; Molnár et al., 2023). Zinc is mainly absorbed as Zn^2+^, but once taken up, it can form stable organometallic complexes with low-molecular-weight exudates containing compounds such as malate, citrate, oxalate, fumarate, acetate, and other organic acids that allow pH reduction (Ajeesh Krishna et al., 2020; Kaur et al., 2024). It has been reported that malic acid exuded by wheat roots was able to mobilize Zn in calcareous soils (Kaur et al., 2024). For Fe, one of the strategies to increase its solubility is rhizosphere acidification, given the formation of ferric oxides and hydroxides, particularly in alkaline and calcareous soils (Molnár et al., 2023).

Under Fe deficiency conditions, plants activate proton pumps (H^+^-ATPases), which are in the plasma membrane of root epidermal cells. These enzymes use ATP energy to expel protons into the rhizosphere, generating acidification in the rhizosphere zone that promotes Fe^3+^ solubilization and facilitates its reduction to Fe^2+^ (Hamzah Saleem et al., 2022). This process is observed in eudicotyledonous and non-grass species, as well as in species such as cork oak (*Quercus suber*), myrobalan plum (*Prunus cerasifera*), and cucumber (*Cucumis sativus*) (Molnár et al., 2023). In addition, roots can release organic acids such as citric, malic, and oxalic acids, as well as phenolic compounds, proteins, and amino acids that can chelate Fe^3+^ and facilitate its mobility in soil (Hamzah Saleem et al., 2022; Molnár et al., 2023). The regulation of these processes is controlled by the transcription factor FIT (FER-like iron deficiency-induced transcription factor), whose expression increases under Fe deficiency, activating genes related to iron acquisition, including those encoding H^+^-ATPases and reductase enzymes (Molnár et al., 2023).

In the case of iodine, only a small fraction remains dissolved in the soil solution; therefore, its availability depends on rhizospheric properties and physicochemical factors that determine the transformation and persistence of iodide (I^-^) and iodate (IO_3_^-^) species in the rhizosphere (Riyazuddin et al., 2023). Unlike Fe and Zn, iodine availability is regulated by its chemical speciation and by microbial transformations at the soil–root interface (Naeem et al., 2026). Microbial communities can reduce iodate to iodide, oxidize iodide to molecular iodine, or generate organic iodine compounds; therefore, concentrations in the rhizosphere are not static and respond to a complex microorganism–soil–plant interaction (Jiang et al., 2024). Additionally, Riyazuddin et al. (2023) highlight that iodine uptake by plants can be stimulated by compounds such as salicylic acid (SA).

Meanwhile, phosphate-solubilizing bacteria can modify Se solubility in the rhizosphere due to the secretion of organic acids that reduce pH. This mechanism is important because selenite shares uptake pathways with phosphate in roots (Deng et al., 2025; Fu et al., 2025).

In summary, rhizosphere acidification, root exudation, and microbial activity modify micronutrient speciation and solubility, controlling nutrient availability and uptake by plants.

### 4.2 Zinc and iron homeostasis: transporters and chelator secretion (strategy I vs. strategy II)

The homeostasis of Zn and Fe depends on a complex network of transporters, chelators, and regulatory mechanisms that allow the balance of adequate micronutrient concentrations to prevent deficiencies and cellular toxicity (Jalal et al., 2024). However, it has also been observed that excess Zn can alter Fe uptake, for example, by inducing physiological symptoms, since both micronutrients share transporters and regulatory pathways, competitive interactions occur during uptake and translocation (Kaur et al., 2024).

For Zn, several transporters are involved in uptake, mobilization, vascular transport, redistribution, and cellular storage. Among them, the ZIP family is particularly important: Zn-Regulated Transporter (ZRT) and Iron-Regulated Transporter (IRT) (Kumar et al., 2016; Pypka et al., 2025; Hacisalihoglu, 2020; Jalal et al., 2024; Ajeesh Krishna et al., 2020). In the case of Fe, Pypka et al. (2025) mention that in *Arabidopsis thaliana*, IRT1 is an Fe^2+^ transporter, although it also transports Zn^2+^. Likewise, IRT2 and IRT3 belong to the ZIP/IRT family and participate in responses related to Fe and Zn; therefore, they share similar mechanisms. In eudicotyledonous plants, Zn acquisition depends mainly on ZIP3 and ZIP5, transporters located in the plasma membrane of the epidermis and root cortex (Robe et al., 2025; Ajeesh Krishna et al., 2020). Under Zn deficiency, plants increase the expression of several ZIP genes, such as IRT3, ZIP1, ZIP3, ZIP4, ZIP5, ZIP9, and ZIP12, with ZIP3 standing out due to its greater functional importance, transporter localization toward the soil, and regulation under conditions of Zn excess (Robe et al., 2025). In monocotyledonous plants, although Zn can be absorbed as free Zn^2+^ or bound to phytosiderophores, evidence from rice indicates that transporters such as OsZIP5 and OsZIP9 are key for Zn^2+^ uptake from the root (Robe et al., 2025; Molnár et al., 2023). Other transporters, such as NRAMP (Natural Resistance-Associated Macrophage Proteins), participate mainly in cellular Zn uptake and mobilization (Gupta et al., 2016).

YSL transporters facilitate Zn transport when it is forming complexes with organic ligands, whereas HMA (Heavy Metal ATPases) transporters are involved in efflux, xylem loading, and internal redistribution (Kumar et al., 2016; Pypka et al., 2025). Finally, the MTP/CDF (Metal Tolerance Proteins/Cation Diffusion Facilitators) families, particularly AtMTP1, participate in vacuolar Zn storage, allowing the maintenance of low free Zn concentrations in the cytosol (Robe et al., 2025). Other families, such as CAX (Cation Exchangers), ZIF (Zinc-Induced Facilitator), and VIT (Vacuolar Iron Transporter), contribute to vacuolar compartmentalization, intracellular storage, and tolerance to metal excess (Jalal et al., 2024; Kumar et al., 2016; Pypka et al., 2025; Hamzah Saleem et al., 2022; Hacisalihoglu, 2020). Likewise, Zn uptake occurs through high-affinity transport systems (HATS) (km = 0.6–2 nM) and low-affinity transport systems (LATS) (km = 2–5 µM) (Hacisalihoglu, 2020; Ajeesh Krishna et al., 2020).

Since it is a charged cation (Zn^2+^), it cannot diffuse freely and therefore requires transporters to enter the cytoplasm (Ajeesh Krishna et al., 2020). Initially, Zn moves by diffusion toward the root cell wall and then through active transport into the cytoplasm via specific proteins (Hacisalihoglu, 2020). Within the root, Zn can be mobilized through apoplastic and symplastic pathways, cross the endodermis, and be loaded into the xylem through HMA2 and HMA4 transporters, which belong to the P1B-type heavy metal ATPases family. These transporters distribute Zn throughout the plant, particularly from roots to shoots (Hamzah Saleem et al., 2022; Kumar et al., 2016).

Meanwhile, the family of Zn- and Fe-regulated transporter proteins has been identified and characterized in prokaryotes, eukaryotes, and archaea; however, they are not completely specific because they can also transport Mn^2+^, Cd^2+^, Co^2+^, Cu^2+^, and Ni^2+^ (Ajeesh Krishna et al., 2020; Robe et al., 2025). Likewise, the presence of phosphorus, boron, copper, and cadmium generates antagonistic interactions that affect Zn uptake and translocation in plants (Martínez-Ríos et al., 2024).

One of the most important chelators is nicotianamine (NA), a non-protein amino acid that forms stable complexes with Zn, Fe, and Cu and participates in intracellular transport. Its expression increases under Zn deficiency conditions, and therefore genes such as *NtZIP4B*, *NtHMA4a/b*, and *NtNAS* may be involved in this process (Pypka et al., 2025). In grasses, nicotianamine can act as a precursor of phytosiderophores from the mugineic acid family (Molnár et al., 2023). In addition, in other hyperaccumulator species, Zn has been found associated with organic ligands such as citrate, malate, and histidine, which allow metal tolerance by facilitating its entry into vacuoles or other cellular compartments (Ajeesh Krishna et al., 2020). Likewise, succinate can form complexes with Zn that decrease its translocation to the aerial parts due to tolerance mechanisms against excess Zn (Kaur et al., 2024).

There are two ways in which plants can acquire Fe and Zn under deficiency conditions, known as strategy I and strategy II. These strategies require the conversion of Fe^3+^ to Fe^2+^ or the transport of Zn²^+^ (the available form for plants) (Molnár et al., 2023). The strategy I mechanism, characteristic of dicotyledonous plants and non-grass monocotyledonous plants, involves the acquisition of Zn and Fe through rhizosphere acidification mediated by H^+^-ATPase pumps (AHA2) (Mattiello et al., 2015; Zhao and Wu, 2017). Another mechanism involves the chelation of citrate, malate, and oxalate to facilitate reduction, as well as electron donation from compounds such as coumarins, flavins, and catechols, which provide electrons for the reduction of Fe^3+^ to Fe^2+^ by the FRO2 enzyme. Fe^2+^ is then absorbed by the IRT1 transporter (Ajeesh Krishna et al., 2020; López-Arredondo et al., 2013; Senguttuvel et al., 2023; Pypka et al., 2025; Molnár et al., 2023), which is regulated by the transcription factor FIT (FER-like Iron Deficiency-Induced Transcription Factor) (Molnár et al., 2023). In strategy II, characteristic of grass species, the mechanism is based on the release of phytosiderophores through transporters such as OsTOM1/OsZIFL4 and the formation of complexes with Fe^3+^ (López-Arredondo et al., 2013; Senguttuvel et al., 2023). In addition, the main synthesized compounds belong to the mugineic acid (MA) family, such as mugineic acid (MA), deoxymugineic acid (DMA), 3-hydroxymugineic acid (HMA), and 3-epi-hydroxymugineic acid (epi-HMA), which form stable Fe^3+^ complexes that can enter the root through proteins of the Yellow Stripe (YS1) family (Ajeesh Krishna et al., 2020; Molnár et al., 2023). Phytosiderophores can also chelate Zn by forming complexes that are absorbed through specific transporters such as Yellow Stripe-Like (YSL) (Mattiello et al., 2015; Zhao and Wu, 2017). The capacity to synthesize phytosiderophores depends on the crop species. For example, barley produces high amounts of MA, HMA, and epi-HMA, whereas maize, rice, and sorghum mainly produce DMA (Molnár et al., 2023). Finally, under deficiency conditions, plants can increase the expression of transporters and activate antioxidant systems to mitigate the stress produced (Mattiello et al., 2015; Zhao and Wu, 2017).

### 4.3 Shared pathways and transporter competition: selenium vs. sulfur, and iodine vs. chloride channels

In the case of selenium, its uptake is closely linked to its similarity with sulfur. Although it depends on the chemical form of Se, selenate (SeO_4_^2-^) is the most bioavailable form, absorbed by roots and transported through vascular bundles via sulfate transporters (Duborska et al., 2022; Sors et al., 2005; Fu et al., 2025). On the other hand, specific Se transporters have not been identified; therefore, Se uptake depends on its chemical species, and selenite utilizes different transport systems associated with sulfate and phosphate, respectively. This explains why Se can compete with essential nutrients through shared metabolic pathways (Deng et al., 2025). This similarity generates competition between Se and S during uptake and assimilation processes, affecting Se dynamics in plants (Duborska et al., 2022). Among the high-affinity sulfate transporters are SULTR1;1, SULTR1;2, and SULTR1;3. However, high sulfate concentrations reduce Se uptake, whereas under S deficiency, Se uptake increases (Deng et al., 2025; Fu et al., 2025). Once incorporated, Se is transported to shoots through the xylem via pathways shared with sulfur (Fu et al., 2025). This transport is mediated by SULTR2;1 (a low-affinity transporter), SULTR1;3, which facilitates selenate transfer and distribution throughout the plant following the pathway from leaves > buds/stems > roots, and finally, SULTR3 transporters, which facilitate sulfate transport into chloroplasts, and SULTR4 transporters, which regulate sulfate release from the vacuole to the cytoplasm. These transporters participate in internal sulfate homeostasis (Deng et al., 2025). In leaves, selenate (SeO_4_^2-^) enters the sulfur assimilation pathway and is converted by ATP sulfurylase into adenosine 5′-phosphoselenate (APSe), which is subsequently reduced to selenite (SeO_3_^2-^) by adenosine 5′-phosphosulfate reductase (Deng et al., 2025). Subsequently, selenite is transformed into selenide (Se^2-^) through sulfite reductase (SiR) or reduced glutathione (GSH), and the resulting selenide is incorporated with O-acetylserine by cysteine synthase to form selenocysteine (SeCys) and selenoamino acids, whose accumulation depends on the developmental stage of the plant (Pickering et al., 2000; Deng et al., 2025; Naeem et al., 2026). On the other hand, selenite is mainly absorbed through phosphate transporters and aquaporins, including PHT1, OsPT, and NIP channels such as OsNIP2;1, which mediate the uptake of selenous acid (H_2_SeO_3_). In crops such as rice, OsPHT1;2 and OsPHT1;8 participate in increasing selenite uptake, whereas in mung bean, PHT1.1 and NIP2 are involved (Deng et al., 2025). However, elevated Se concentrations can induce toxicity, causing oxidative stress, reduced growth, and metabolic alterations due to the substitution of sulfur in proteins (Schiavon et al., 2016; Terry et al., 2000; Deng et al., 2025).

Although specific iodine transporters have not yet been identified, iodine appears to depend on H^+^/anion symporter transport systems, chloride channels, and other anion transport systems, following pathways like those of chloride (Silva-Herrera et al., 2025; Riyazuddin et al., 2023; Duborska et al., 2022). Iodine (I) is mainly taken up as iodide (I^-^), where iodide uses anionic transport systems shared with chloride because both are monovalent halides with similar physicochemical properties (White et al., 2009; De Angeli et al., 2006; Silva-Herrera et al., 2025). Although the evidence is indirect, several Cl^-^ transporters may participate in iodide movement. These include the NPF family, such as ZmNPF6.4 and ZmNPF6.6, which mobilize protons that transport anions across the plasma membrane; the CLC, CCC, and ALMT families, which participate in anion homeostasis; as well as SLAH1/SLAH3 channels, involved in Cl^-^ loading into the xylem, which could represent potential pathways for iodide translocation (Silva-Herrera et al., 2025).

Once inside the plant, iodine moves slowly through the xylem; however, transport through the phloem has also been observed in species such as tomato and lettuce (Riyazuddin et al., 2023). After its uptake by the plant, iodine is incorporated into metabolic pathways leading to the formation of various organic compounds, including iodosalicylates, iodobenzoates, iodotyrosine and plant analogues of thyroid hormones (Rakoczy-Lelek et al., 2021; Smoleń et al., 2021). Although iodine is not essential for most plants, at low concentrations it can improve growth and tolerance to salinity stress through the activation of antioxidant enzymes and osmotic adjustment (Kiferle et al., 2021; Leyva et al., 2011). However, high iodine concentrations can cause toxicity, manifested as chlorosis and leaf necrosis (Mackowiak et al., 2005). Iodate generally causes lower phytotoxicity than iodide because its uptake depends on a metabolic step requiring prior reduction to I^-^ through reductase enzymes present in roots (Riyazuddin et al., 2023). Smoleń et al. (2021) mention that iodide (I^-^) could be oxidized before uptake through vanadium-dependent haloperoxidase enzymes (vHPO), generating hypoiodous acid (HOI), which facilitates iodine transfer and its entry into the root.

### 4.4 Biological interactions: the role of plant growth-promoting bacteria and arbuscular mycorrhizal fungi in the rhizosphere

One of the important mechanisms that promote micronutrient uptake and homeostasis in plants is the biological interaction in the rhizosphere, where plant growth-promoting bacteria (PGPB) and arbuscular mycorrhizal fungi (AMF) modify the rhizospheric microenvironment through nutrient transformation and mobilization, the production of bioactive metabolites, the modulation of root physiology, and, in the case of AMF, the expansion of the soil volume explored through their hyphal networks (Jain et al., 2020; Kumar et al., 2025; Jalal et al., 2024; Founoune-Mboup et al., 2024; Upadhayay et al., 2022; Jiang et al., 2024).

Among the genera frequently reported as PGPB are *Bacillus*, *Pseudomonas*, *Azospirillum*, *Azotobacter*, *Enterobacter*, *Burkholderia*, *Klebsiella*, *Rhizobium*, and *Gluconacetobacter*, which improve mineral nutrition through biological nitrogen fixation, phytohormone production, synthesis of siderophores and amino acids, nutrient solubilization through organic acid production, and suppression of phytopathogens (Jalal et al., 2024; Upadhayay et al., 2022). Among the relevant mechanisms are zinc-solubilizing bacteria (ZSB), which secrete organic acids such as gluconic acid, 2-ketogluconic acid, citric acid, and lactic acid, allowing rhizosphere acidification and the solubilization of insoluble Zn forms, such as ZnO, ZnCO_3_, and Zn_3_(PO_4_)_2_, through chelation reactions (Jalal et al., 2024; Upadhayay et al., 2022). Other bacteria, such as *Burkholderia cepacia*, can solubilize Zn through oxalic acid, formic acid, tartaric acid, and acetic acid. Likewise, species such as *Microbacterium saperdae*, *Enterobacter cancerogenes*, and *Pseudomonas monteilii* produce metallophores or chelating compounds that increase Zn bioavailability in soil (Upadhayay et al., 2022; Jalal et al., 2024).

Arbuscular mycorrhizal fungi form symbiotic associations with more than 80% of terrestrial plants and perform essential functions. Through the hyphal networks they generate, AMF expand soil exploration within the rhizosphere (Founoune-Mboup et al., 2024). Furthermore, AMF symbiosis improves Fe homeostasis in plants within the hyphosphere, the zone composed of fungal hyphae, through increased root size and branching, enhanced root exudation, and interactions with soil bacterial communities. Additionally, Fe is required for fungal hyphal development, which promotes the release of low-molecular-weight compounds such as oxalic and citric acids that solubilize Fe and influence the efficiency of the AMF–plant symbiosis (Rajapitamahuni et al., 2023; Founoune-Mboup et al., 2024).

In plants that employ strategy I mechanisms based on rhizosphere reduction and acidification through H^+^ extrusion mediated by epidermal H^+^-ATPases, AMF can enhance rhizospheric acidification, promoting Fe^3+^ solubilization, increasing the expression of *IRT1* and *FRO2* genes, and improving photosynthetic parameters (Kabir et al., 2020; Chao and Chao, 2022). In addition, fungi can acquire Fe through direct Fe^2+^ uptake, peroxidase/permease systems, heme/hemin acquisition, and siderophore-mediated pathways, although these mechanisms remain under investigation (Molnár et al., 2023; Rajapitamahuni et al., 2023). In *Rhizophagus irregularis*, candidate genes involved in Cu, Fe, and Zn transport have been identified, including a putative ferrireductase (*RiFRE1*), iron permeases (*RiFTR1* and *RiFTR2*), multicopper oxidase genes (*RiMCO1–RiMCO9*), and NRAMP-type transporters (*RiSMF1*, *RiSMF2*, *RiSMF3.1*, and *RiSMF3.2*), which may participate in Fe acquisition and homeostasis (Rajapitamahuni et al., 2023). In grasses, AMF can release phytosiderophores and induce the expression of genes associated with Fe–phytosiderophore chelation and transport, such as *SbDMAS2*, *SbNAS2*, and *SbYS1* in sorghum. However, responses are species-dependent; for example, in maize, genes such as *ZmNAS1* and *ZmYS1* showed higher expression in non-mycorrhizal plants (Rajapitamahuni et al., 2023). Regarding Fe and Zn nutrition, Founoune-Mboup et al. (2024) demonstrated that inoculation with *Glomus mosseae* in pearl millet (*Pennisetum glaucum* L.) combined with cattle or horse manure increased foliar concentrations of Fe, Zn, P, and K. AMF can modify Fe chelation strategies; in grasses, they stimulate phytosiderophore release, the expression of genes associated with DMA, NAS, and YSL transporters, as well as the formation of Fe–phytosiderophore complexes within the plant (Founoune-Mboup et al., 2024). For Zn, AMF contribute through immobilization within fungal hyphae, vacuolar storage in fungal structures, complexation with polyphosphates, and deposition in glomalin-related structures, thereby regulating Zn phytotoxicity (Kaur et al., 2024).

For Se, soil microorganisms participate in Se speciation through the reduction of selenate (SeO_4_^2-^) and selenite (SeO_3_^2-^) into elemental selenium (Se^0^), mediated by biological reducing agents such as glutathione, bacillithiol, hydrogen sulfide, and siderophores through specific pathways (Deng et al., 2025). In shallot (*Allium cepa* var. *aggregatum*), AMF inoculation increased selenocystine concentration by 36% and selenate concentration by 21% compared with non-inoculated plants (Schiavon et al., 2020). Sojka et al. (2026) and Deng et al. (2025) indicate that the solubilization of selenate and selenite into more assimilable organic forms depends on AMF and plant growth-promoting bacteria, highlighting genera such as *Glomus/Funneliformis*, *Bacillus*, *Pseudomonas*, and *Comamonas*. Regarding Se, Qin et al. (2023) demonstrated that in rice (*Oryza sativa* L. cv. Xinliangyou 6), inoculation with *Funneliformis mosseae* combined with sodium selenite (Na_2_SeO_3_) did not reduce mycorrhizal colonization and increased biomass, Se concentration, and Se accumulation efficiency, while reducing antagonistic interactions between Se and elements such as Ni, Mo, P, and Cu. During early stages of Se deficiency stress, plants may recruit Se-reducing bacteria, whereas in later stages they may recruit Se-methylating microorganisms that promote volatilization and reduce Se translocation to aerial tissues (Deng et al., 2025).

For iodine, the plant–rhizosphere microbiome interaction remains poorly studied; however, evidence indicates that bacteria can reduce, oxidize, accumulate, and volatilize different iodine species. These microorganisms can use IO_3_^-^ as a terminal electron acceptor, including *Pseudomonas* sp. SCT, *Denitromonas* sp. IR-12, and *Shewanella oneidensis* MR-1, which reduce iodate to iodide using acetate and lactate as electron donors. These processes are mediated by specialized enzymatic complexes such as IdrABP1P2, DmsEFAB, and MtrCAB (Jiang et al., 2024). Riyazuddin et al. (2023) indicate that iodine is associated with soil organic matter, highlighting microbial methylation processes that produce methyl iodide (CH_3_I), which can be released into the atmosphere and reincorporated into soils through atmospheric deposition. Furthermore, S and Fe cycles are linked to iodine through the generation of H_2_S and Fe^2+^, which participate in the reduction of oxidized iodine species. Meanwhile, iodide-oxidizing bacteria use multicopper oxidases such as IoxA and IoxC to oxidize iodide into molecular iodine (I_2_), I_3_^-^, and other organoiodine compounds (Jiang et al., 2024).

Bacteria associated with AMF, such as *Bacillus*, *Streptomyces*, *Enterobacter*, *Rhizobium*, and *Microbacterium*, can produce siderophores and promote Fe transfer to plants through the hyphal network. Additionally, genes related to siderophore production have been detected in AMF species such as *Gigaspora rosea*, *Rhizophagus irregularis*, *Glomus cerebriforme*, and *Diversispora epigea* (Rajapitamahuni et al., 2023; Founoune-Mboup et al., 2024). Furthermore, some mycorrhiza-helper bacteria stimulate spore germination, hyphal branching, and root colonization, thereby enhancing nutrient acquisition (Rajapitamahuni et al., 2023). Interactions between PGPB and AMF are generally synergistic. Bacteria can function as mycorrhiza helper bacteria (MHB), stimulating spore germination, hyphal growth and development, as well as mycorrhizal colonization, which contributes to nutrient solubilization, uptake, transport, and availability (Founoune-Mboup et al., 2024).

## 5 Vascular transport, internal redistribution, and subcellular homeostasis

### 5.1 Long-distance transport: xylem loading and phloem mobility

Long-distance transport of the micronutrients Zn, Fe, I, and Se depends on the chemical form absorbed, the transporters involved, the capacity of the element to be solubilized and mobilized, as well as xylem loading and phloem mobility (Ajeesh Krishna et al., 2020).

After entering the root symplast, Zn can be immobilized in root vacuoles or transported cell-to-cell through plasmodesmata toward the vascular bundles. Subsequently, Zn is exported to the apoplastic xylem and transported to shoots in chelated forms, thereby preventing Zn adsorption to cell walls (Sinclair and Krämer, 2012). During transport, Zn^2+^ crosses the Casparian strip, the endodermis, and xylem parenchyma cells. At this point, HMA2 and HMA4 transporters, belonging to the P-type ATPase family, load Zn into the xylem from parenchyma cells (Ajeesh Krishna et al., 2020). The transport of Zn^2+^ from roots to aerial tissues occurs mainly through the xylem (pH ≈ 5.5), driven by transpiration flow, whereas phloem loading of Zn is mediated by HMA transporters located in the pericycle and xylem parenchyma (Hamzah Saleem et al., 2022; Pypka et al., 2025). Zinc transported through the phloem is directed toward developing sink organs, including tubers, grains, and shoots (Hamzah Saleem et al., 2022). In grains, Senguttuvel et al. (2023) reported that Zn accumulates mainly in the aleurone layer and subsequently moves toward the inner endosperm through vascular tissues, whereas Fe remains associated with phytic acid. Mentewab et al. (2023) reported that when the Fe–citrate pathway is inhibited, plants rely on alternative pathways such as Fe–nicotianamine complexes to facilitate Fe loading into the xylem. Pypka et al. (2025) indicated that *NtHMA4a/b* encode xylem transporters, whereas *NtNAS* encodes nicotianamine synthase, which is involved in nicotianamine synthesis. Hu et al. (2023) reported that during critical stages of sink organ filling and senescence, leaves can function as source organs by exporting Zn required for developing tissues through the phloem.

Zhu et al. (2022) showed that Fe can be loaded into the xylem as Fe^2+^; however, for translocation, it requires the formation of Fe^3+^–citrate complexes supported by the ferroxidases LPR1 and LPR2, which facilitate the oxidation of Fe^2+^ to Fe^3+^ and regulate Fe levels in xylem cell walls. Ueda (2025) demonstrated that, in rice, OsFDRL1 is a citrate transporter in the xylem that facilitates Fe transport, although excessive Fe accumulation may induce toxicity symptoms. Mentewab et al. (2023) highlighted that Fe transport does not depend on a single pathway but rather on a network of transport systems, including Fe–citrate pathways involving citrate released by FRD3 and Fe–nicotianamine (Fe–NA) complexes transported by WBC19. Senguttuvel et al. (2023) emphasized that OsYSL9 is a Fe-chelate transporter that translocates Fe from the endosperm to the embryo during rice seed development. Additionally, genes such as *OsVIT1* and *OsVIT2* participate in reducing Fe sequestration in vacuoles. Zhu et al. (2022) showed that reduced expression of transporters such as LPR1 and LPR2 increases the proportion of Fe^2+^ in xylem sap, promoting Fe deposition in vascular bundles and altering Fe distribution in younger leaves, which ultimately results in chlorosis.

For iodine (I), the molecular transport system within xylem vessels remains unclear. Nascimento et al. (2022) explained that iodine follows pathways like chloride (Cl^-^), involving H^+^/anion symporters, channels, H^+^/anion antiporters, tonoplast anion channels, and organic acid transporters. However, iodine can also be absorbed through aerial structures such as stomata and the cuticle. Within plants, iodine can accumulate in vacuoles and follows an accumulation pattern from roots < leaves < stems (Nascimento et al., 2022). However, it can also be volatilized as CH_3_I through HMT/HTMT enzymes (Kiferle et al., 2021). Li et al. (2016) reported that I accumulates preferentially in organs with higher transpiration rates and observed the following distribution pattern: roots > stems > fruits, suggesting that iodine is transported mainly through the xylem. Smoleń et al. (2021) indicated that, in addition to inorganic forms such as I^-^ and IO_3_^-^, other aromatic organic iodine compounds should be considered in plant tissues, including 5-iodosalicylic acid (5-ISA) and 3,5-diiodosalicylic acid (3,5-diISA). In lettuce, these compounds showed higher uptake and translocation efficiency from roots to leaves following the order: 5-ISA > 3,5-diISA > KIO_3_. These compounds can be transformed into iodine, salicylic acid, iodotyrosine, and plant thyroid hormone-like compounds such as T3 and T4.

For Se, Zhang and Chu (2022) and Trippe and Pilon-Smits (2021) reported that selenate enters plants through sulfate transporters such as SULTR1;1, SULTR1;2, and SULTR1;3, and is transported toward the xylem. In contrast, selenite can enter through phosphate transporters such as OsPHT1;2 (OsPT2) and aquaporins such as OsNIP2;1, after which it can be transformed into SeMet. During senescence, SeMet is translocated to grains through amino acid transporters, including OsAAP1, OsAAP3, and OsNRT1.1B. Trippe and Pilon-Smits (2021) also indicated that transporters belonging to the SULTR3 family may participate in selenate import into chloroplasts, whereas SULTR2;1 and SULTR3;5 contribute to selenate translocation from roots to shoots. Long-distance transport forms, sink allocation, and reported physiological thresholds are summarized in **Table 3**.

### 5.2 Cellular assimilation, metabolic functions, and coping with trace element toxicity

In the case of Zn, plants are considered zinc-deficient when leaf Zn concentrations are below 15–20 mg Zn kg^-1^ dry weight (DW) (Ajeesh Krishna et al., 2020). Optimal zinc levels in leaves range between 30 and 100 mg Zn kg^-1^ DW (White and Broadley, 2011). Concentrations above this range, specifically between 100 and 700 mg Zn kg^-1^ leaf DW, may cause zinc toxicity, manifested as chlorosis, reduced growth, and oxidative stress (Kaur and Garg, 2021).

Cellular zinc transport is mediated in part by the MTP/CDF, which contributes to cellular detoxification. When MTP3 expression is high in cortical and epidermal cells, Zn mobility from roots to aerial tissues is restricted, promoting its sequestration in vacuoles (Hamzah Saleem et al., 2022). In roots, Zn storage is regulated by chelating proteins (Gupta et al., 2016). Within the cytoplasm, Zn does not remain as a free ion because it can bind to macromolecules and induce toxicity; therefore, it is maintained in chelated forms by low-molecular-weight ligands and possibly by plant metallothioneins (Sinclair and Krämer, 2012).

In leaves, Zn accumulates mainly in vacuoles, trichomes, and epidermal cells, and can be redistributed from source or senescent tissues toward meristems, young leaves, inflorescences, and seeds through the phloem (Sinclair and Krämer, 2012).

When Zn availability is heterogeneous in soils, this element can be translocated from Zn-sufficient roots to Zn-deficient tissues, reducing deficiency responses and limiting further Zn uptake. This process involves modifications in the expression of genes such as *NtZIP4B* and *NtZIP5B*, which are associated with Zn import; *NtHMA4a/b*, involved in Zn export; and *NtNAS*, related to nicotianamine-mediated chelation (Pypka et al., 2025). Conversely, Zn deficiency promotes Zn remobilization from older leaves to younger tissues (Li et al., 2022). Kaur et al. (2024) reported that Zn excess tolerance is mediated through the formation of glutathione (GSH) and phytochelatin (PC) complexes.

Gao et al. (2023) reported that Zn deficiency affects photosynthesis in rice, particularly in the active reaction centers of photosystem II (PSII; RC/ABS), as Zn plays an enzymatic role associated with organelles such as chloroplasts and mitochondria. It is also involved in CO_2_ maintenance in mesophyll cells, antioxidant defense, and the metabolism of proteins and nucleic acids (Hu et al., 2023; Kumar et al., 2025). On the other hand, Kumar et al. (2025) indicated that total Zn concentrations in plants are maintained within the micromolar range, explaining why plants avoid accumulating free Zn, as excessive Zn levels can cause chlorosis, reduced growth, and oxidative stress.

Regarding Fe, in flooded soils with low pH, Fe^3+^ is reduced to soluble Fe^2+^, and this excess Fe can be absorbed by roots and transported to the xylem with the support of the citrate transporter OsFRDL1, resulting in oxidative damage in shoots (Aung and Masuda, 2020). Iron is present in all components of the chloroplast electron transport chain, with up to 80% of total Fe located in chloroplasts. Therefore, Fe deficiency can cause chlorosis, reduce the P700 reaction center activity, and inhibit photosynthesis (Gao et al., 2023). Additionally, free Fe^2+^ can participate in the Fenton reaction, generating hydroxyl radicals (•OH) and reactive oxygen species (ROS), which damage membranes, DNA, and cellular structures, leading to leaf bronzing, reduced growth, and even plant death (Nascimento et al., 2022). Consequently, Aung and Masuda (2020) described four defense mechanisms against Fe excess: suppression of Fe uptake and transport genes, Fe retention in roots, and ROS detoxification mechanisms. The activation of Fe transporters can also induce Zn accumulation in roots, where plants activate detoxification mechanisms involving vacuolar transporters such as MTP3 and HMA3, as well as transporters such as ZIF1, which contribute to Zn sequestration and storage within cells (Sinclair and Krämer, 2012).

For iodine (I), iodate is generally less phytotoxic than iodide because it must be reduced before being absorbed by plants (Duborska et al., 2022; Kiferle et al., 2021). Nascimento et al. (2022) explained that iodine can accumulate in vacuoles, where it acts as a cellular buffer. Kiferle et al. (2021) identified iodinated proteins associated with PSII, PSI, cytochrome b6f, ATPase, and Rubisco, suggesting that iodine has physiological and antioxidant defense functions. Iodine is mainly stored in its inorganic form as I^-^; however, it can also occur as IO_3_^-^ and as different organic iodinated compounds (Senguttuvel et al., 2023). Nascimento et al. (2022) reported that iodine-containing compounds include iodosalicylates, iodobenzates, monoiodotyrosine, diiodotyrosine, and triiodothyronine, which accumulate in vacuoles, where iodine is passively transported and stored.

For selenium (Se), toxicity is associated with its incorporation into proteins in the forms of selenocysteine (SeCys) and selenomethionine (SeMet). Selenium hyperaccumulator plants avoid this toxicity by redirecting SeCys toward non-protein compounds such as methylselenocysteine (MeSeCys), γ-glutamyl-methylselenocysteine (γ-GGMeSeCys), and selenocystathionine (Sors et al., 2005). Trippe and Pilon-Smits (2021) indicated that one of the tolerance mechanisms involving the conversion of SeCys to MeSeCys is mediated by the enzyme selenocysteine methyltransferase (SMT). At the cellular level, Se can be stored in plastids as SeCys and SeMet, which may either be incorporated into proteins or volatilized (Nascimento et al., 2022).

### 5.3 Comparative synthesis of shared and nutrient-specific mechanisms

Although Zn, Fe, Se, and I differ markedly in chemical form and biological function, their behavior across the soil–plant continuum is governed by several shared control points. For all four micronutrients, soil pH, redox conditions, organic matter, mineral surfaces, rhizosphere processes, and nutrient–nutrient interactions influence the fraction available for plant acquisition. Plants further regulate acquisition through changes in the rhizosphere, transporter activity, chelation, vascular loading, redistribution, and intracellular sequestration. These common control points explain why soil availability alone does not necessarily predict accumulation in edible organs (Ajeesh Krishna et al., 2020; Hamzah Saleem et al., 2022; Pypka et al., 2025).

Important nutrient-specific differences arise from their chemical forms and transport pathways. Zn and Fe are predominantly acquired as cations or metal–ligand complexes and are regulated by overlapping transporter and chelator networks, including ZIP/IRT, YSL, HMA, NRAMP, MTP, VIT, citrate, and nicotianamine pathways. This overlap creates both coordination and competition between Zn and Fe during uptake, xylem loading, redistribution, and cellular storage (Ajeesh Krishna et al., 2020; Zhu et al., 2022; Mentewab et al., 2023; Pypka et al., 2025). By contrast, Se and I largely exploit transport systems evolved for chemically analogous nutrients. Selenate is mainly transported through sulfate transporters, whereas selenite can enter through phosphate-related transporters and aquaporins; iodine appears to use anion transport systems related to chloride, although specific iodine transporters remain poorly characterized (Zhang and Chu, 2022; Riyazuddin et al., 2023; Silva-Herrera et al., 2025).

The elements also differ in their principal physiological constraints. Zn and Fe require strict homeostatic regulation because excess free metal ions can disrupt cellular metabolism, whereas Se toxicity is strongly influenced by chemical form and the substitution of sulfur in proteins, and excess I can cause growth inhibition, chlorosis, and necrosis. Therefore, biofortification strategies should not treat Zn, Fe, Se, and I as functionally equivalent targets. Instead, successful multi-nutrient biofortification requires combining shared management principles with element-specific knowledge of speciation, transport, remobilization, storage, and toxicity (Schiavon et al., 2016; Terry et al., 2000; Riyazuddin et al., 2023; Wang et al., 2025; Akhter et al., 2026).

### 5.4 Physiological bottlenecks limiting micronutrient accumulation in edible organs

Efficient root uptake does not necessarily translate into high micronutrient concentrations in edible organs. After acquisition, nutrients must pass several physiological control points, including root retention, xylem loading, long-distance transport, transfer between vascular tissues, phloem remobilization, unloading into sink organs, and intracellular storage. Limitations at any of these steps can restrict biofortification even when soil or foliar nutrient supply is adequate (White and Broadley, 2011; Hamzah Saleem et al., 2022; Mentewab et al., 2023).

For Zn, root vacuolar sequestration can restrict movement toward shoots, while remobilization from vegetative tissues and nodes determines the final delivery to reproductive sinks. Evidence from rice illustrates this limitation: enhanced OsHMA2-mediated Zn movement toward reproductive tissues was accompanied by substantial Zn retention in node I, indicating that increased uptake or leaf remobilization alone may not guarantee efficient grain loading (Hu et al., 2023). Similar constraints apply to Fe, for which citrate- and nicotianamine-mediated transport, xylem chemistry, YSL-dependent redistribution, and vacuolar sequestration influence delivery to developing seeds and edible tissues (Zhu et al., 2022; Mentewab et al., 2023; Senguttuvel et al., 2023).

For Se and I, physiological bottlenecks are additionally shaped by the transport systems they share with sulfate, phosphate, and chloride, respectively, as well as by chemical speciation and tissue-specific mobility. Se accumulation depends on the balance between uptake, assimilation into Se-containing amino acids, remobilization, and detoxification, whereas I generally shows limited phloem mobility and its internal distribution is strongly affected by chemical form and species-specific transport capacity (Zhang and Chu, 2022; Riyazuddin et al., 2023; Wang et al., 2025; Akhter et al., 2026).

Finally, increased mineral concentration in an edible organ does not necessarily imply improved nutritional value. Localization within non-edible tissues, storage in poorly remobilized compartments, dilution associated with high yield, and antinutritional compounds such as phytic acid can reduce the fraction ultimately available for human absorption. Therefore, successful biofortification should evaluate not only total micronutrient concentration, but also nutrient allocation to edible tissues, chemical form, bioaccessibility, antinutrient interactions, and yield stability (Gibson et al., 2018; Xue et al., 2023; Ram et al., 2024).

## 6 Implications for crop biofortification strategies: toward biofortified futures

Biofortification has emerged as an agricultural and public health strategy to address hidden hunger and micronutrient deficiencies among vulnerable populations. This approach aims to increase the concentration, bioaccessibility, and bioavailability of micronutrients in the edible parts of crops (Ofori et al., 2022; Shohael et al., 2025). This strategy is particularly relevant in contexts where diets rely heavily on staple crops with low micronutrient content, such as rice, wheat, maize, potato, common bean, and cowpea, and where access to diverse diets remains limited. FAO et al. (2022) reported that by 2021, more than 3 billion people were unable to afford a healthy and lower-cost diet, while as many as 828 million people experienced hunger in the same year. In this context, biofortification represents a cost-effective and sustainable intervention, with estimates suggesting that every USD invested may generate up to USD 17 in economic and social benefits (Mkambula et al., 2022; Stangoulis and Knez, 2022).

The urgency of developing “biofortified futures” is supported by the global magnitude of Zn, Fe, I, and Se deficiencies (Ofori et al., 2022). Zinc deficiency affects approximately 17–31% of the global population. It is associated with stunted growth, diarrhea, increased susceptibility to infections, immune dysfunction, DNA damage, and a higher risk of chronic diseases (UNICEF/WHO/World Bank, 2021; Upadhayay et al., 2022; Senguttuvel et al., 2023). Iron deficiency also represents a critical global burden: up to 80% of the population may have insufficient Fe intake, approximately 30% may suffer from anemia due to chronic Fe deficiency, and this condition is associated with around 0.8 million deaths annually (Midya et al., 2021; Senguttuvel et al., 2023). Likewise, micronutrient deficiencies are linked to infant and maternal mortality, impaired cognitive development, reduced physical work capacity, and increased healthcare burdens. Ofori et al. (2022) reported that 1.1 million of the 3.1 million annual child deaths are attributed to micronutrient deficiencies. Therefore, increasing Zn, Fe, I, and Se concentrations in staple foods represents not only an agronomic challenge but also a public health priority.

This issue becomes more complicated as a result of the decline in mineral concentration in plant foods caused by the adoption of crops that focus on high yields but ignore nutrients combined with the higher efficiency of plants in using nutrients which causes a dilution effect on mineral content in plant tissues (Marles, 2017). Shohael et al. (2025) show that Ca content has decreased by 29%, Mg by 21%, K by 6%, P by 11%, and Fe by 32% between 1963 and 1992 and that by 2019, Na has decreased by 52%, Fe by 50%, Cu by 49%, and Mg by 10%. This problem is aggravated by the process of milling, in which mineral-rich components like aleurone layer and embryo of wheat, or bran and germ of rice are removed (Koç and Karayiğit, 2021). Therefore, biofortification should be understood not only as a response to current deficiencies but also as a preventive strategy against future declines in the nutritional quality of crops.

From a strategic perspective, biofortification can be achieved through three main approaches: agronomic biofortification, conventional breeding, and transgenic or biotechnological approaches, which are currently complemented by omics technologies, gene editing, and tools such as CRISPR-Cas9 (Ofori et al., 2022; Koç and Karayiğit, 2021). Agronomic biofortification enables a rapid response through the application of fertilizers to the soil, foliar sprays, seed treatments, biofertilizers, chelates, nanofertilizers, or beneficial microorganisms. However, its effectiveness depends on soil nutrient availability, pH, organic matter content, nutrient interactions, applied chemical form, dosage, plant developmental stage, the capacity for translocation into edible plant tissues, as well as on the presence and expression of specific or non-specific transporters (Ramzan et al., 2020; De Valença et al., 2017; Kromann et al., 2017; White et al., 2012).

Genetic and biotechnological biofortification aims to increase micronutrient concentration and bioavailability through germplasm selection, conventional breeding, transgenesis, gene editing, and manipulation of genes involved in mineral uptake, transport, storage, and redistribution (Younas et al., 2022; Sharma et al., 2020). In rice, Zaman et al. (2017) indicated that Zn availability can be improved by combining agronomic management with genetic improvement. In contrast, Velu et al. (2014) highlighted that genetic and agronomic biofortification approaches are complementary strategies for increasing Zn and Fe concentrations in wheat.

Several studies have demonstrated positive effects from combining agronomic biofortification with genetic improvement, representing an efficient strategy for reducing Zn and Fe deficiencies in staple crops (Gómez-Coronado et al., 2016; Kromann et al., 2017; Swamy et al., 2016; Zevallos et al., 2024).

### 6.1 Translating soil-plant interactions into agronomic biofortification

Agronomic biofortification translates soil–plant continuum into management practices that increase micronutrient concentrations in edible plant tissues through soil fertilization, foliar application, seed treatments, biofertilizers, chelates, and nanofertilizers. **Table 4** summarizes the key crops, test environments, fertilizer types, application rates, application methods, and outcomes reported for agronomic biofortification. The effectiveness of agronomic biofortification is influenced by several factors such as the type of mineral used, application rate, material solubility, soil pH, organic matter in the soil, application timing, crop species and origin, as well as the presence of antinutritional compounds such as phytic acid, which can reduce micronutrient bioavailability (De Valença et al., 2017; Younas et al., 2022; Cakmak et al., 2010; Ramzan et al., 2020).

As pointed out by Upadhayay et al. (2022), agronomic biofortification works in conjunction with genetic strategies when the abundance of Zn limits its accumulation inside plants. The use of fertilizers enriched with micronutrient elements, for instance, zinc sulfate, provides greater zinc levels in edible tissues and enhances growth and crop yield. This becomes particularly pressing when one considers that the lack of zinc is responsible for the decline of photosynthesis, carbohydrate production, RNA, and protein metabolism, which, in its turn, ultimately influences the efficiency and quality of crops. Thus, Zn fertilization serves as a tool for the management of agricultural crops.

Silva et al. (2025) demonstrated that Zn agronomic biofortification in Brazil must consider fertilizer source, cost, and soil conditions. In a PRISMA systematic review including 24 studies published from 2014 to 2024, they reported that ZnSO_4_·7H_2_O is the most used Zn source due to its high solubility (570–1000 g L^-1^ at 20 °C), effectiveness in acidic soils, and low cost. In contrast, Zn-EDTA is more suitable for alkaline soils because of its stability up to pH 7–10, although it may cost up to four times more. This comparison highlights that fertilizer selection should be adapted to soil properties, crop requirements, and economic feasibility.

#### 6.1.1 Foliar fertilization

Advantages of using foliar Zn application over soil fertilization have become apparent depending on the crops used. Certain crops, such as wheat, showed a significant increase in grain Zn levels upon this application. For foliar application, several Zn sources have been used, including ZnSO_4_, Zn(NO_3_)_2_, and Zn-EDTA. Among these, ZnSO_4_ has been shown to be effective in alleviating Zn deficiency. Moreover, the efficacy of foliar application depends oncrop species, environmental conditions, and the composition of the fertilizer. Young plant tissues are characterized by greater nutrient_uptake owing to their intense growth. Moreover, foliar application may help preserve soil fertility as well as increase yield of potato tubers (Hussain et al., 2022; Zhao and Wu, 2017; Velu et al., 2014; Cakmak et al., 2010; Stomph et al., 2011; Ierna et al., 2020).

Foliar application of ZnSO_4_·7H_2_O has emerged as an efficient strategy to increase Zn concentration without compromising crop yield. Across four locations in China, Zhang and Chu (2022) observed that 0.4% ZnSO_4_·7H_2_O increased Zn concentration in whole wheat grain by up to 58%. Sánchez-Palacios et al. (2023) reported that foliar ZnSO_4_ applications ranging from 0.4% to 0.5%, or 5–15 mM, applied during flowering, flag leaf development, or grain filling stages, can increase grain Zn concentrations from 25–40 mg kg^-1^ to 35–80 mg kg^-1^. The highest increases were observed with two foliar applications in soils containing 0.91–1.2 mg kg^-1^ DTPA-extractable Zn (Zhang and Chu, 2022; Sánchez-Palacios et al., 2023). Gutierrez-Vilchez et al. (2026) evaluated agronomic Zn biofortification in potato across six fields located in Peru, under arid coastal conditions. Foliar application of 5 kg Zn ha^-1^ increased Zn concentration in the flesh by up to 40%. However, foliar applications appeared to influence productivity. Hussain et al. (2022) reported significant differences among potato cultivars following foliar application of 10 g L^-1^ Zn in alkaline and calcareous soils.

Oliveira et al. (2024) provided experimental evidence from cowpea grown in an acidic Typic Quartzipsamment soil with low Zn-DTPA availability. During the 2021–2022 growing seasons, 20 genotypes were evaluated with and without the application of 600 g Zn ha^-1^ as ZnSO_4_·7H_2_O applied at full flowering. All genotypes showed increased yield following Zn application. Foliar Zn application increased leaf Zn concentration up to seven-fold, approximately doubled grain Zn concentration, and increased total amino acids and storage proteins, including albumin, globulin, prolamin, and glutelin fractions.

Foliar biofortification can also enrich underground storage organs with other micronutrients. Rakoczy-Lelek et al. (2021) demonstrated in carrot (*Daucus carota* L.) cv. Octavo F1, grown in Poland, that four foliar applications between BBCH 16 and BBCH 42 with 200–400 g I ha^-1^ and 5–20 g Se ha^-1^ increased I and Se concentrations without affecting yield or commercial quality. Iodine was applied as I^-^ and selenium as SeO_4_^2-^. Although iodine accumulated mainly in leaves, it was also translocated to storage roots. Selenium concentration increased in both leaves and roots; however, Se concentration in roots was 4.5-fold lower than that in leaves.

Selenium fertilization requires particular attention due to its narrow range between deficiency and toxicity. Agronomic Se doses are generally low, approximately 10–20 g Se ha^-1^, applied either to the soil or through foliar sprays as selenate or selenite. However, only approximately 12% of soil-applied Se is absorbed by plants, whereas foliar application can be up to eight times more efficient. Furthermore, some genes and proteins, e.g., ATP sulfurylase (APS), adenosine 5′-phosphosulfate reductase (APR), and selenocysteine methyltransferase (SMT), play an important role in the regulation of Se build-up, tolerance, and distribution in plant tissues (Senguttuvel et al., 2023; Sors et al., 2005).

Wang et al. (2017) compared six forms of foliar Se fertilization in wheat and maize: sodium selenite, sodium selenate, selenomethionine (SeMet), nano-Se, humic acid + selenite, and compound fertilizer + selenite. The application rates were 30 g Se ha^-1^ for wheat and 60 g Se ha^-1^ for maize. Foliar application increased grain Se concentration by 0.02–0.31 mg kg^-1^ in wheat and by 0.07–1.09 mg kg^-1^ in maize compared with the control. Selenium recovery efficiency ranged from 3.0–10.4% in wheat and 4.1–18.5% in maize, with selenate showing the highest efficiency and nano-Se the lowest.

In wheat, selenite resulted in grain Se concentrations that were 6–138% higher than those obtained with other Se forms; whereas in maize, selenate generated Se concentrations 1–2 times higher than those obtained with Se(IV), SeMet, or nano-Se. Although nano-Se is considered a stable form with a lower risk of toxicity, nano-Se particles with a size of 142.8 ± 9.1 nm resulted in the lowest Se concentrations and recovery efficiencies. This was attributed to the fact that cell wall pores are generally smaller than 5 nm, limiting nanoparticle penetration and translocation within plant tissues (Wang et al., 2017).

According to Xue et al. (2023), using nanofertilizers is sometimes inferior to traditional fertilizers. The use of low dose Zn nanofertilizers did not result in any significant increase in performance, and in some instances, the traditional ZnSO_4_ was found to be better than its nanoparticle version. This suggests that the efficiency of foliar biofortification depends on the source of the nutrient, amount of application, form, plant physiology, variety and environmental conditions as well.

Foliar fertilization also enables the development of multi-nutrient biofortification strategies. Zou et al. (2019) evaluated foliar applications of I, Zn, and a cocktail containing I, Zn, Se, and Fe in wheat across six countries. Grain Zn concentration increased from 28.6 to 46.0 mg kg^-1^ with 0.5% Zn applied as ZnSO_4_·7H_2_O and reached 47.1 mg kg^-1^ with the cocktail solution. In wheat cultivated in India and Pakistan, Se was applied as 0.001% Na_2_SeO_4_ within a cocktail containing 0.5% ZnSO_4_·7H_2_O and 0.04% KIO_3_, demonstrating the effectiveness of selenate in increasing grain Se concentration (Zou et al., 2019; Duborska et al., 2022).

#### 6.1.2 Soil fertilization

Kour et al. (2025) confirmed that Zn-enriched fertilizers can improve grain mineral concentration and potential Zn bioavailability. In the reviewed studies, soil-applied Zn increased grain Zn concentration by 23% in maize, 7% in rice, and 19% in wheat, whereas foliar Zn application increased Zn concentration by 30%, 25%, and 63%, respectively. Furthermore, Zn application reduced phosphorus and phytate concentrations in grains, suggesting enhanced Zn bioavailability.

Greenhouse studies on wheat found that the application of 5 mg Zn kg^-1^ using ZnO and ZnSO_4_ increased grain Zn concentration by 58% and 30%, respectively (Almutari et al., 2025) whereas the application of Zn enriched compost (9 mg Zn kg^-1^) resulted in a 60% increase; in the field, a rate of 18 kg Zn ha^-1^ led a 63% increase compared to the control (Abbas et al., 2021). Another field study using a rate of 12 kg Zn ha^-1^ increased grain Zn concentration by 15% (Rafique et al., 2026).

Sánchez-Rodríguez et al. (2017) demonstrated that Zn biofortification must consider P–Zn interactions, particularly in calcareous Vertisols with low Zn-DTPA availability. In 20 soils from southern Spain, with Zn-DTPA concentrations ranging from 0.20 to 0.58 mg kg^-1^ and Olsen P values between 2.6 and 31.3 mg kg^-1^, wheat grain Zn concentration showed a negative correlation with Olsen P (R² = 0.501; *P* < 0.001). The application of 50 mg P kg^-1^ reduced grain Zn concentration from 20.4 to 12.0 mg kg^-1^, whereas the addition of 2 mg Zn kg^-1^ increased it to 36.8 mg kg^-1^. Furthermore, P application increased the P/Zn ratio from 125 to 279, indicating a stronger antagonistic effect between these nutrients.

Jalal et al. (2024) reported that Zn application can enhance Fe uptake up to a certain threshold, as excessive Zn levels may reduce Fe uptake in wheat. In a study conducted in alkaline soils of Pakistan under wheat cultivation, Zn application up to 8 kg ha^-1^ increased total Zn uptake, as well as Mn uptake, and enhanced Fe uptake; however, higher Zn rates resulted in reduced Fe uptake (Abbas et al., 2009).

Zn-humic acid complexes also form slow-release sources of Zn, ensuring that enough of the elements are available to plants and losses of Zn due to immobilization or precipitation are reduced. Consequently, the organic matter of the soil determines the chemical form of Zn present in the soil solution, minimizes losses of Zn from the soil, and facilitates its release over time. Thus, this method combines fertilizer management and soil chemistry with the stable supply of micronutrients to plants (Boguta and Sokolowska, 2016).

#### 6.1.3 Seed priming

Among the different biofortification strategies addressed in this review, seed treatment, also known as seed priming, has gained increasing attention. This technique consists of controlling hydration of seeds up to the onset of Phase II, during which physiological and metabolic processes are activated prior to radicle emergence (Tripathi et al., 2024; Sahoo et al., 2025). Primed seeds exhibit more synchronous emergence, greater early vigor, and improved root system development, which enhances nutrient acquisition and is particularly important during the early stages of plant development. In addition to improving initial crop establishment, seed priming requires relatively low doses of the applied compounds, making it a complementary strategy to soil and foliar applications within agronomic biofortification programs (García-Locascio et al., 2024). Depending on the agent used for seed pre-treatment, seed priming can be classified as hydropriming (water), halopriming (inorganic salts), osmopriming (osmotic agents), nutripriming (nutrient solutions), and biopriming (beneficial microorganisms) (Paparella et al., 2015).

Several studies have already reported the effects of seed priming on different widely consumed crops. In rice seeds, combined Zn and Se pre-treatment has been shown to exert a synergistic effect, providing greater benefits than the individual application of either element. This combination promoted synchronous emergence and greater seedling vigor, while also increasing chlorophyll, protein, and phenolic compound contents, as well as nutrient uptake (Adhikary et al., 2022). Similarly, combined Zn and N treatment in rice seeds improved germination, seedling vigor, and root growth during the early stages of development, while increasing Zn concentration in young tissues. However, these effects of seed priming were not sustained until crop maturity. In contrast, foliar application of Zn and N during later developmental stages increased yield and promoted Zn accumulation in the grain, with increases of up to 37.9% compared with the control. Recent results in wheat and barley also indicate that seed priming with Fe, Zn, and B promotes plant growth and root development during the early stages of crop development and may mitigate the effects of water deficit, particularly when micronutrients are applied individually. However, these positive effects were not fully reproduced under field conditions, where favorable climatic conditions and limited replication may have masked the benefits of the treatments (Iglesias-Ganado et al., 2025).

#### 6.1.4 Use of microorganisms

Studies have demonstrated that bacterial inoculation can increase micronutrient accumulation. For example, *Pseudomonas fluorescens* increased Zn concentration by 33–35% in maize shoots and by 37–42% in maize grains; *Enterobacter cloacae* subsp. *dissolvens* increased Zn by 37% and Fe by 21% in wheat grains; and *Exiguobacterium aurantiacum* MS-ZT10 produced increases of up to six-fold in Fe and Zn accumulation in wheat (Upadhayay et al., 2022). In broccoli, Se volatilization to dimethyl selenide (DMSe), considered a Se detoxification mechanism, decreased by 95% when roots were treated with antibiotics such as penicillin-G and chlortetracycline, highlighting the role of microorganisms in Se transformation processes (Duborska et al., 2022). Malathi et al. (2024) evaluated the inoculation of *Enterobacter cloacae* ZSB14, a Zn-solubilizing bacterium, in calcareous soil (pH = 8.47, 12.0% CaCO_3_, 0.59 mg kg^-1^ DTPA-extractable Zn). The inoculation reduced soil pH to 7.66 and consequently increased Zn availability, reaching 1.78 mg kg^-1^ after 90 days of incubation. Upadhayay et al. (2022) reported that Zn-solubilizing microorganisms, such as *Trichoderma harzianum* and *Bacillus amyloliquefaciens*, can induce the expression of ZIP family genes (ZRT/IRT).

Research by Jain et al. (2020) reported that Serratia sp. strains ZTB15, ZTB24, ZTB28, and ZTB29 successfully control Zn levels in Zn-polluted soils from the Zawar mining region of Rajasthan by promoting Zn immobilization via exopolysaccharide (EPS) production as rhizospheric buffers. At the same time, resistance mechanisms such as CZC transporters, P-type ATPases, and metallothionein proteins are utilized for Zn detoxification by means of biosorption, bioprecipitation, and bioaccumulation (Jain et al., 2020; Kaur et al., 2024).

Hamzah Saleem et al. (2022) state that Zn-solubilizing bacteria such as *Enterobacter*, *Bacillus*, and *Pseudomonas* enhance Zn availability through the acidification of rhizosphere and production of organic acids. It has also been shown that *Exiguobacterium aurantiacum* strain MSZT10 increases Zn and Fe concentrations by up to 6 times in Triticum aestivum grains. Furthermore, *Bacillus halotolerans*, *Enterobacter hormaechei*, and *Pseudomonas frederiksbergensis* have been reported to promote seed germination, plant growth, and Zn uptake.

### 6.2 Genetic and biotechnological breeding: targeting transporters and enhancing micronutrient bioavailability in edible organs

Conventional breeding and recombinant DNA technology through genetic engineering are applied to increase nutrient bioavailability, nutrient concentration, and nutrient uptake in staple foods, a strategy known as genetic biofortification, which can improve nutritional quality (Sharma et al., 2020). Conventional breeding is still widely used in some parts of the world; although it is a long-term approach, it has the potential to be cost-effective and represents a politically feasible pathway in several regions (Sharma et al., 2020; Kumari et al., 2022). This viable alternative allows the crossing of nutrient-rich parental lines with promising agronomic traits by exploiting available genetic variability, including varieties capable of hybridization with wild relatives (Younas et al., 2022; Sharma et al., 2020). This indicates that conventional breeding techniques could reduce antinutrient levels and promote the accumulation of compounds that enhance the absorption of essential nutrients (Velu et al., 2014). Akhtar et al. (2019) highlighted the limited potential of cereal genotypes for Zn uptake and the restricted transport of Zn within plants due to its interaction with phytate. Some efforts have been reported by Guttieri et al. (2006) to minimize phytate concentrations (0.52–4.56 mg g^-1^) in staple crops such as maize, rice, and wheat.

In rice, Upadhayay et al. (2022) demonstrated wide variability among genotypes for agronomic traits, Zn, and Fe concentrations, with moderate to high heritability for several traits, indicating that a significant proportion of this variation can be exploited through conventional breeding. Similarly, Senguttuvel et al. (2023) reported broad ranges of Zn and Fe concentrations in rice: Zn varied from 6.2 to 71.6 mg kg^-1^ in brown rice and from 4.8 to 40.9 mg kg^-1^ in polished rice, whereas Fe ranged from 6.9 to 67.3 mg kg^-1^ in brown rice and from 0.8 to 36.45 mg kg^-1^ in polished rice. This confirms that genotype strongly influences the final accumulation of micronutrients (Upadhayay et al., 2022; Senguttuvel et al., 2023).

In a GWAS (genome-wide association study) involving 166 wheat varieties from four areas in China, Tong et al. (2022) discovered that the Zn concentrations measured between 21.8 and 58.9 mg kg^-1^, while the Fe concentrations were recorded as being between 32.5 and 74.6 mg kg^-1^. The heritability index was found to be 0.71 and 0.72 for Zn and Fe, respectively, confirming high heritability in the study. In addition, the study also identified 41 loci that were linked to accumulation, with a maximum of 25 loci associated with Zn and 16 loci linked with Fe; hence, out of those loci, 17 loci and eight loci were classified as possible QTLs that are not known yet.

According to Pypka et al. (2025), soil spatial heterogeneity plays a vital role in Zn homeostasis, and so do the genes responsible for Zn transport and redistribution in plants. For example, *NtZIP4B* profiles in *Nicotiana tabacum* plants grown under a heterogeneous Zn regime for 14 days showed that the *NtZIP4B* deficiency profile in the roots prevailed in 70% of roots cultivated under Zn depletion conditions while the sufficient profile was noted in 90% of roots grown under Zn over-sufficiency conditions. These suggest that importers and exporters of Zn, as well as nicotianamine-type chelators, are responsible for integrating Zn status signals in the whole plant.

The role of OsHMA2 transporter in Zn loading and mobilizing during rice plant senescence was described by Hu et al. (2023). The *OsHMA2* gene was strongly upregulated in the node I leaf, suggesting that its encoded transporter contributes to Zn transport from source tissues into the phloem. However, the data obtained by the authors also show that the increase of foliar mobilization of Zn is not enough and a significant part remains deposited in node I thus making it one of the key barriers in Zn delivery to the grains. Thus, OsHMA2 transporter and associated regulators are involved in senescence and nodal transport.

The authors Shohael et al. (2025) describe that using genetic engineering techniques to change genes contributing to nutrient uptake, transport, chelation, and storage leads to increased concentrations of Fe and Zn in rice and wheat. The implementation of ferritin genes from common bean and pearl millet together with OsNAS2 gene in rice resulted in Fe concentration in endosperm reaching levels very close to HarvestPlus goals at about 13 µg g^-1^ Fe and 28 µg g^-1^ Zn. This could help meet 30% of the daily requirements.

Genetic biofortification is a technology that holds great potential for potato crops but is challenging because of their complex genetics and genotype-environment interactions. Seid et al. (2023) evaluated 45 tetraploid genotypes biofortified by the International Potato Center (CIP) in Holetta, Ethiopia, revealing significant variation in Fe, Zn, yields, and tuber quality. The iron content ranged from 14.68 mg kg^-1^ to 26.07 mg kg^-1^ dry weight, while zinc levels ranged from 10.22 mg kg^-1^ to 20.59 mg kg^-1^. The genotype CIP312767.014 had the highest iron concentration of 26.07 mg kg^-1^ and 19.14 mg kg^-1^ Zn content, which is nearly 50% higher than the commercial variety ‘Gudanie’. Zinc showed great broad sense heritability of 64.86% and a significant expected genetic increment, whereas iron showed a moderate heritability of 40.20%. In addition, the positive correlation between Fe and Zn (r = 0.68) suggests that both minerals may be improved together.

Agrawal et al. (2024) further reported that potato biofortification can be accomplished through genetic and biotechnological approaches, including conventional breeding, marker-assisted selection, QTL mapping, tissue-specific transgenesis, and CRISPR-Cas9 technology. For example, in the CIP-HarvestPlus program, Fe-rich and Zn-rich diploid Andean native potatoes were crossed with disease-resistant tetraploid clones, as a result of which Fe and Zn levels were increased from 0.48 mg Fe 100 g^-1^ and 0.35 mg Zn 100 g^-1^ to 0.73 mg Fe 100 g^-1^ and 0.63 mg Zn 100 g^-1^ respectively within three rounds of breeding.

The role played by the environment must be considered in ways to increase the concentrations of Zn in grains using biofortification. Khokhar et al. (2024) indicated that the new varieties with the ability to accumulate more Zn in grains do not lead to the expected increase in the concentration of Zn in grains due to the limitations posed by the soil conditions. Biofortified varieties from programs such as HarvestPlus can increase the concentration of Zn in grains by some 8–12 mg kg^-1^ but their actual performance is influenced by soil conditions, soil pH, and agronomic practices. This position is supported by Velu et al. (2014), who emphasized that soil Zn availability is a limiting factor despite improvements in Zn uptake or mobilization.

Phytate restricts the availability of both Zn and Fe in cereals, which indicates that elimination of antinutrients or enrichment with the compatible chelators can raise the real nutritional value of crops. However, the accumulation of toxic materials such as Cd or Pb should also be avoided because some transporters of micronutrients can also retrieve chemically similar metals (Akhtar et al., 2019; Guttieri et al., 2006; Cakmak et al., 2010; Qaswar et al., 2017; Hussain et al., 2019).

### 6.3 Integrated approaches to mitigate global micronutrient deficiencies

Integrated biofortification approaches aim to combine genetic improvement, agronomic management, multi-nutrient fertilization, bioavailability assessment, environmental adaptation, and social adoption to reduce global micronutrient deficiencies. This perspective is necessary because increasing mineral concentration in crops alone does not guarantee nutritional impact, as other factors such as yield, trait stability, human absorption, antinutrients, processing, cost, cultural acceptance, and implementation policies also influence the outcome.

Ram et al. (2024) demonstrated the synergy between genetic and agronomic biofortification in Zn-biofortified wheat in India and Pakistan, grown on alkaline/calcareous soils with low DTPA-Zn availability (0.42–0.59 mg kg^-1^; Gujranwala 0.71 mg kg^-1^). The combination of biofortified genotypes, with 50 kg ZnSO_4_·7H_2_O ha^-1^ applied to soil, and foliar applications of Zn, I, and Se increased grain Zn concentration by up to 62.7% in India and 43% in Pakistan. Additionally, the Zn+I+Se cocktail increased Fe by 19% in Pakistan and Se by 106% in India and 123% in Pakistan. Although slight yield reductions were observed in India with the cocktail and Zn+I treatments, this strategy provided more than 15 mg kg^-1^ additional Zn and enabled grain Zn concentrations above 50 mg kg^-1^ (Ram et al., 2024).

According to Xue et al. (2023), it is necessary to factor in aspects such as mineral concentrations, yield, bioavailability, nutrient interactions, and dilution effects in integrated biofortification programs. In maize, foliar application of ZnSO_4_ or a micronutrient combination improved the grain Zn amounts by 17.6 mg kg^-1^ in ZD958, 10.8 mg kg^-1^ in DH605, and 8.7 mg kg^-1^ in LD510, although neither of them had Zn (31.2 mg kg^-1^) and Fe (32.5 mg kg^-1^) concentrations that met the thresholds set by HarvestPlus. The authors of the study found a positive relationship between Zn and Fe (r = 0.849), a decrease in the amount of phytic acid by 6.3% to 10.6%, lowered PA/Zn and PA/Fe ratios, as well as an increase in daily Zn consumption. However, a negative correlation between yield on the one hand and Zn, Fe, N, Mn, Cu, and Mg concentrations on the other has indicated a dilution effect due to higher yields.

According to Upadhayay et al. (2022), it is essential to take an integrated approach to rice growing since biofortification should not only aim for raising Zn and Fe levels but should also secure high yield, good grain quality, and proper agronomic adjustment. In addition, it is possible to combine genetic biofortification with the application of ZnSO_4_ fertilizers if Zn is limited in soil and thus prevents micronutrient accumulation in grains. The study conducted by Tong et al. (2022) reported that wheat biofortification with Zn and Fe is based on integration of genetics, environment, and agronomic characteristics.

Garg et al. (2018) argue that biofortification should combine conventional breeding, agronomic practices, and biotechnology because each strategy addresses different limitations. Zevallos et al. (2024), in a soil with pH 7.88, low soil organic matter (1.48%), CaCO_3_ content of 1.86%, and high available P (39 mg kg^-1^), found that these conditions limited Zn solubility in the soil despite high total Zn content. Therefore, foliar Zn fertilization increased tuber Zn concentration through the integration of genetic and agronomic biofortification in potato using five genotypes: four biofortified clones and the commercial variety ‘Yungay’. Foliar Zn application showed no genotype × dose interaction for yield components, although significant differences among genotypes were observed. In contrast, tuber Zn concentration showed a highly significant genotype × fertilization interaction: biofortified clones increased Zn concentration by an average of 43% (28–61%), even under alkaline soil conditions, whereas ‘Yungay’ increased by only 6%. These results demonstrate that the combination of conventional breeding and foliar Zn fertilization can act synergistically to enhance Zn accumulation in potato tubers.

Praharaj et al. (2021) emphasized that biofortification should consider Zn concentration, yield, bioavailability, consumer acceptance, soil environment, and economic benefits. In staple grains, only approximately 25% of Zn is estimated to be bioavailable, mainly due to antinutrients such as phytic acid; however, phytates and polyphenols also have beneficial roles in human health and plant physiology. Therefore, breeding strategies should reduce limitations to nutrient absorption without eliminating their positive functions. Additionally, agronomic Zn biofortification of wheat could reduce up to 56.6% of the health burden associated with Zn deficiency, with costs ranging from USD 226–594 per DALY averted through foliar spraying alone and USD 41–108 when combined with pesticide applications (Praharaj et al., 2021).

Koç and Karayiğit (2021) proposed that integrated biofortification should consider fertilization, micronutrient recycling, soil microbiology, and environmental sustainability. Khokhar et al. (2024) support the integration of genetic and agronomic approaches by demonstrating that genetic potential is not fully expressed when soil conditions limit Zn availability. In 328 farmer fields in Ethiopia, wheat grain Zn concentration ranged from 11.8 to 63.9 mg kg^-1^, with a median of 25.3 mg kg^-1^ and a six-fold variation influenced by soil organic carbon and pH.

Abdu et al. (2022) presented biofortification as an integrated approach connecting agriculture, nutrition, and public health. Their discussion deals not only with the increase of crop Zn concentration but also with dietary intake, bioavailability, and disease burden, calculated as Disability-Adjusted Life Years (DALYs). It seems that agronomic Zn biofortification, especially foliar application in Ethiopia is a feasible option to decrease Zn deficiency in children. The impact of this approach will depend on agronomic, economic, and logistical issues, and therefore it should be combined with soil diagnostics, crop types, infrastructure, cost of implementation, and identification of target population.

Mineral accumulation in tubers does not translate into enhanced nutrition for humans. For potatoes, factors influencing Zn accumulation include its genetic potential and transport to tubers, as well as restrictions caused by compounds such as phytic acid, which are known for its high affinity for Zn and Fe. Phytates accumulate in roots, tubers, stems, and leaves, where they have been reported to constitute about 40% of total P. This leads us to conclude that phytate/Zn and phytate/Fe ratios are useful parameters for determining bioavailability and actual nutritional needs (Hawkesford et al., 2011; Gibson et al., 2018).

White et al. (2017) demonstrated that nutritional impact should be evaluated based on absorbable Zn rather than total Zn alone. In potato cultivar ‘Saxon’, consumption of a 200 g portion of biofortified potato increased Zn intake from 1.04 to 1.58 mg, although phytate content also increased from 15.0 to 21.6 μmol flour^-1^. This reduced the estimated Zn bioavailability from 24.2% to 22.27%; however, absorbable Zn increased by 11.4%. This increase could reduce the proportion of adults with low Zn intake in the United Kingdom by up to 12%.

Jongstra et al. (2020) highlighted the importance of integrating biofortification with dietary assessment in humans. In Peru and Malawi, regular potatoes contained 0.33 mg Fe 100 g^-1^, whereas biofortified potatoes contained 0.69 mg Fe 100 g^-1^; in sweet potato, values were 0.55 and 0.97 mg Fe 100 g^-1^, respectively. In women with ferritin ≤25 μg L^-1^, consuming 500 g of biofortified potato could provide approximately 33% of the daily Fe requirement, compared with 18% from biofortified orange-fleshed sweet potato. These results suggest that biofortified potato can improve Fe intake, although the presence of inhibitors such as polyphenols should also be considered (Jongstra et al., 2020).

Liria-Domínguez et al. (2023) provided direct evidence of human Zn absorption using stable isotopes. In 37 women who consumed 500 g of regular and biofortified potatoes, biofortified potatoes contained 0.48 ± 0.02 mg Zn 100 g^-1^ fresh weight, compared with 0.32 ± 0.03 mg Zn 100 g^-1^ in regular potatoes. The absorption fraction can differ because of the structure of the food but absorbed Zn in the case of biofortified potatoes was found to be considerably higher, giving the result of 0.49 ± 0.13 mg per 500 g portion. Therefore, it proves that biofortification affects not only Zn concentration but also the level of Zn absorption in general.

The success of biofortification programs will depend on a comprehensive understanding of micronutrient interactions along the soil–plantsoil–plant–food continuum, as well as the integration of knowledge about plant physiology, micronutrient bioaccessibility, and human nutrition. This integrated approach will be fundamental for designing sustainable biofortification strategies.

## 7 Concluding remarks

Addressing the global burden of hidden hunger requires a fundamental shift from solely maximizing crop yields to prioritizing nutritional density in crop production. As synthesized in this review, the success of crop biofortification with zinc (Zn), iron (Fe), selenium (Se), and iodine (I) depends entirely on mastering the multi-level mechanisms governing the soil–plant continuum. Micronutrient mobility and availability are not static soil properties; they are dynamically regulated by parent material, texture, pH, redox potential, soil organic matter, and complex nutrient-nutrient interactions. Plants actively respond to these edaphic constraints by modifying the rhizosphere, including proton extrusion, exudation of organic acids and phytosiderophores, and functional symbioses with plant growth-promoting bacteria and arbuscular mycorrhizal fungi to solubilize and mobilize essential elements.

Overcoming physiological bottlenecks requires targeting specific molecular pathways that regulate nutrient homeostasis. While the acquisition and transport of Zn and Fe depend on dedicated transporter networks, Se and I use pathways shared with essential nutrients such as sulfate, phosphate, and chloride. Developing effective biofortification strategies requires addressing these distinct pathways, along with internal constraints including xylem loading, phloem mobility to sink organs, vacuolar sequestration, and antinutritional factors such as phytic acid, which diminishes human bioavailability.

Despite substantial advances, important knowledge gaps remain. The mechanisms controlling the final allocation of micronutrients to edible organs are still incompletely understood, particularly those regulating phloem remobilization, nodal transfer, sink strength, and tissue-specific storage. Iodine transport remains less well characterized than that of Zn, Fe, and Se, and the contribution of specific anion transporters to I uptake and redistribution requires further validation. For Se, the narrow range between beneficial and toxic concentrations, together with strong species- and chemical-form dependence, continues to complicate the definition of broadly applicable agronomic thresholds. Future studies should consider not only total micronutrient concentration but also its localization and chemical form within edible tissues, antinutritional compounds, processing losses, bioaccessibility, and, where possible, human bioavailability. Increases in total tissue concentration do not necessarily translate proportionally into greater absorbable nutrient intake, emphasizing the need to extend evaluation beyond plant mineral concentration alone. Future research should also prioritize the validation of mechanistic responses under contrasting field conditions, with particular emphasis on genotype × environment × management interactions and multi-location, multi-season experiments. In parallel, multi-nutrient studies should quantify synergistic and antagonistic interactions among Zn, Fe, Se, and I rather than optimizing each micronutrient independently. Single-track approaches are inherently limited. Agronomic biofortification, using targeted soil amendments, foliar sprays, seed priming, biofertilizers, and nanofertilizers, provides immediate increases in nutrient levels in grain and other edible parts. Yet, its efficiency remains heavily constrained by soil chemistry and local edaphic factors. Conversely, genetic biofortification, marker-assisted selection, and biotechnological tools deliver sustainable, long-term genetic gains. Still, these fortified cultivars cannot reach their full potential in nutrient-depleted or chemically restrictive soils.

Consequently, the future of biofortification rests on integrated, multi-nutrient strategies. Combining precise soil diagnostic frameworks, tailored agronomic management, beneficial rhizospheric microorganisms, and elite crop varieties engineered for high nutrient uptake and tissue-specific allocation provides a robust blueprint for biofortified food systems. Bridging agricultural productivity, environmental sustainability, and public health will ensure that biofortified crops deliver high yields, consumer-acceptable quality, and bioavailable nutrition to vulnerable populations worldwide.
